# Advancing polytrauma care: developing and validating machine learning models for early mortality prediction

**DOI:** 10.1186/s12967-023-04487-8

**Published:** 2023-09-25

**Authors:** Wen He, Xianghong Fu, Song Chen

**Affiliations:** 1 Reproductive Medicine Center, Quzhou People’s Hospital, The Quzhou Affiliated Hospital of Wenzhou Medical University, No. 100, Minjiang Avenue, Quzhou, 324000 Zhejiang China; 2 Department of Orthopedics, Quzhou People’s Hospital, The Quzhou Affiliated Hospital of Wenzhou Medical University, No. 100, Minjiang Avenue, Quzhou, 324000 Zhejiang China

**Keywords:** Polytrauma, Mortality, Random forest, Neural network, XGBoost

## Abstract

**Background:**

Rapid identification of high-risk polytrauma patients is crucial for early intervention and improved outcomes. This study aimed to develop and validate machine learning models for predicting 72 h mortality in adult polytrauma patients using readily available clinical parameters.

**Methods:**

A retrospective analysis was conducted on polytrauma patients from the Dryad database and our institution. Missing values pertinent to eligible individuals within the Dryad database were compensated for through the k-nearest neighbor algorithm, subsequently randomizing them into training and internal validation factions on a 7:3 ratio. The patients of our institution functioned as external validation cohorts. The predictive efficacy of random forest (RF), neural network, and XGBoost models was assessed through an exhaustive suite of performance indicators. The SHapley Additive exPlanations (SHAP) and Local Interpretable Model-Agnostic Explanations (LIME) methods were engaged to explain the supreme-performing model. Conclusively, restricted cubic spline analysis and multivariate logistic regression were employed as sensitivity analyses to verify the robustness of the findings.

**Results:**

Parameters including age, body mass index, Glasgow Coma Scale, Injury Severity Score, pH, base excess, and lactate emerged as pivotal predictors of 72 h mortality. The RF model exhibited unparalleled performance, boasting an area under the receiver operating characteristic curve (AUROC) of 0.87 (95% confidence interval [CI] 0.84–0.89), an area under the precision-recall curve (AUPRC) of 0.67 (95% CI 0.61–0.73), and an accuracy of 0.83 (95% CI 0.81–0.86) in the internal validation cohort, paralleled by an AUROC of 0.98 (95% CI 0.97–0.99), an AUPRC of 0.88 (95% CI 0.83–0.93), and an accuracy of 0.97 (95% CI 0.96–0.98) in the external validation cohort. It provided the highest net benefit in the decision curve analysis in relation to the other models. The outcomes of the sensitivity examinations were congruent with those inferred from SHAP and LIME.

**Conclusions:**

The RF model exhibited the best performance in predicting 72 h mortality in adult polytrauma patients and has the potential to aid clinicians in identifying high-risk patients and guiding clinical decision-making.

**Supplementary Information:**

The online version contains supplementary material available at 10.1186/s12967-023-04487-8.

## Introduction

Trauma reigns as the foremost contributor to mortality and disability globally, with over 5 million fatalities per year stemming from incidents such as falls, vehicular collisions, landslides, and explosions. Polytrauma patients predominantly contribute to this statistic, representing 65% to 72% of cases [1, 2]. These individuals frequently endure grievous injuries, concomitant with hemorrhagic or traumatic shock and immune dysregulation, necessitating precise evaluation and expeditious intervention. Therefore, the prompt identification of patients susceptible to in-hospital fatalities is vital for safeguarding patient well-being, judiciously allocating medical resources, and curtailing healthcare expenditures [3].

Machine learning algorithms have demonstrated great potential in predicting medical outcomes and complications, aiding clinicians in making informed decisions, and enhancing patient care [4–6]. Moreover, recent advancements in machine learning techniques, from traditional linear models to complex deep learning architectures, have shown the ability to handle large, heterogeneous datasets and capture intricate relationships among variables that may not be apparent using conventional statistical approaches [7–9]. This informs the crafting of a precise and dependable prognostic model to discern the elevated risk of early mortality in polytrauma patients and empowers healthcare practitioners to pinpoint such individuals, thereby facilitating the enactment of tailored preventative strategies and remedial actions.

The objective of this study is to develop and validate a machine learning-driven prognostic model for assessing the risk of mortality within 72 h post-admission in multi-trauma patients, utilizing an assorted array of patient attributes and clinical determinants. We aim to compare the performance of various machine learning algorithms in terms of their predictive accuracy and clinical utility. Ultimately, we seek to provide a valuable reference for clinicians that can assist in the early identification of polytrauma patients at risk of early death and facilitate the implementation of targeted preventive strategies to diminish the prevalence thereof.

## Methods

**Fig. 1:**
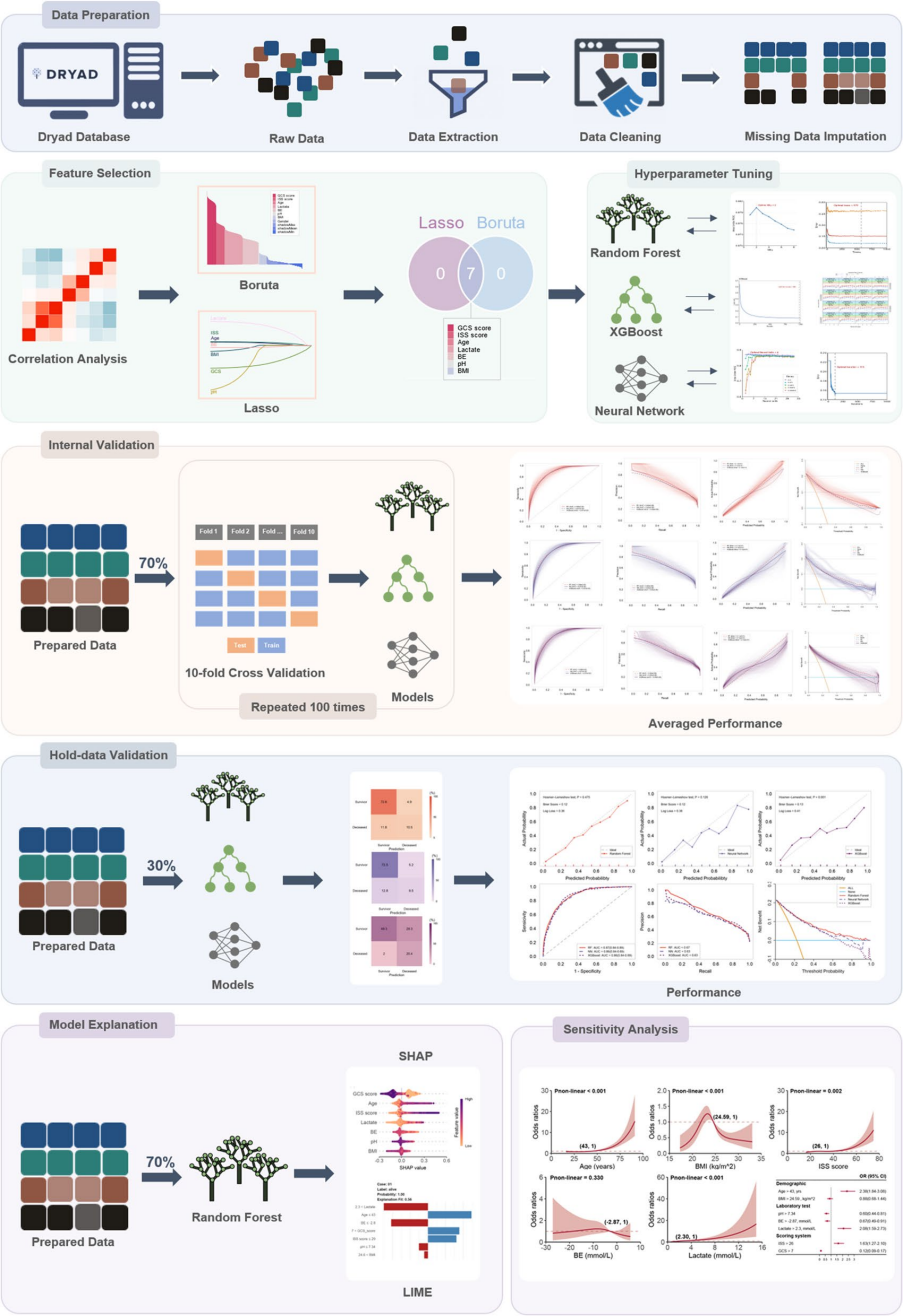
Schematic of the study workflow. *Lasso* least absolute shrinkage and selection operator, *GCS* Glasgow Coma Scale, *ISS* injury severity score, *BE* base excess, *BMI* body mass index, *SHAP* shapley additive explanations, *LIME* local interpretable model-agnostic explanations

This study followed the reporting guidelines of the STROBE guidelines. The overall workflow chart was illustrated in Fig. 1.

### Data source

Datasets for model training and internal validation can be accessed through the Dryad Digital Repository [10]. The Dryad, an open resource database, offers a diverse assortment of discoverable, liberally reusable, and citable research data. Confidential information within the repository has been rendered anonymous. Data acquisition adheres to the tenets delineated in the Declaration of Helsinki and has received local ethics committee endorsement.

To externally validate the prediction model, medical records of patients who had been admitted to the Quzhou Affiliated Hospital of Wenzhou Medical University (Zhejiang, China) were retrospectively analyzed. The study protocol was approved by the institutional review boards of Swissethics Kantonale Ethikkommision Zu¨ruch (Approval Number: [KEK-Zu¨rich]) [10] and the Quzhou Affiliated Hospital of Wenzhou Medical University (Approval Number: [2023CL060]), which waived the need to obtain patient informed consent.

### Study design and participants

Based on the cohort study performed by Halvachizadeh et al. [10], which encompassed multi-injured adult patients (> 18 years old) receiving care at the University Hospital of Zurich’s Level I trauma center between January 1, 1996, and January 1, 2013, whilst excluding individuals afflicted by chronic ailments, neoplastic diseases, or genetic abnormalities impacting the musculoskeletal framework. The time from injury to admission was delineated as less than 24 h. Patients sustaining multiple traumas were defined using an Injury Severity Score (ISS) of 16 or greater in conjunction with the Berlin definition criteria [11]. Variables selected from the dataset for analysis were summarized in Table 1, including patient demographics, scoring information, and laboratory data, such as age, gender, body mass index (BMI), ISS, glasgow coma scale (GCS), pH, base excess (BE), and lactate. The laboratory data were the first values obtained upon admission. The outcome was ascertained as the patient's demise within 72 h post-admission. Pertinent variable measurements have been meticulously delineated in the previous publication [10]. Ultimately, of the 3668 recorded patients, 3075 were enrolled, discounting 579 (15.8%) with ISS values less than 16, 13 (0.4%) lacking outcome data, and 1 (0.03%) with an erroneous body mass index (BMI) value denoted as 0 (Additional file 1: Figure S1).

**Table 1:** Baseline characteristics of training, internal, and external validation sets

	Training set (N = 2153)	Internal validation set (N = 922)	External validation set (N = 1673)	*P* Value^c^
Demographic characteristics				
Age^a^, years	43 (28, 61)	43 (29, 60)	42 (28, 58)	0.106
Sex^b^				0.482
Female	555 (25.8)	257 (27.9)	441 (26.4)	
Male	1598 (74.2)	665 (72.1)	1232 (73.6)	
BMI^a^, kg/m^2	24.6 (23.3, 26.0)	24.6 (23.4, 26.0)	24.5 (22.4, 26.3)	0.339
Scoring system				
ISS^a^	29 (22, 38)	27 (22, 38)	27 (22, 35)	0.076
GCS^a^	7 (3, 15)	7 (3, 14)	13 (3, 15)	** < 0.001**
Laboratory test				
pH^a^	7.34 (7.28, 7.37)	7.34 (7.28, 7.38)	7.34 (7.29, 7.38)	**0.020**
BE^a^, mmol/L	− 2.80 (− 5.40, − 1.15)	− 2.98 (− 5.40, − 1.15)	− 2.67 (− 4.90, − 1.00)	**0.028**
Lactate^a^, mmol/L	2.30 (1.50, 3.40)	2.30 (1.50, 3.54)	2.20 (1.50, 3.20)	**0.042**

Bold values indicate a *p*-value of less than 0.05

*BMI* *body mass index*, *ISS* injury severity score, *GCS* glasgow coma scale, *BE* base excess

^a^Values are presented as the median (inter-quartile range)

^b^Values are presented as number (percentage)

^c^*P* values between groups were assessed by the Chi-square and Kruskal–Wallis H tests

The external validation datasets were retrospectively extracted from 2,289 patients who met the aforementioned inclusion and exclusion criteria in our institution between January 1, 2017, and March 1, 2023. Patient selection is described in detail in Additional file 1: Figure S1.

### Missing data

The percentage of missing data from the Dryad database is presented in Additional file 1: Figure S2. To optimize statistical power and minimize bias, the k-nearest neighbor (KNN) [12] imputation with k equal to 10 was used to impute missing values in eligible patients. The comparisons between the raw data and imputation data were illustrated in Additional file 1: Figure S3. In order to eliminate differences in the distribution of BMI between the imputation and raw data, a new round of imputation of missing values for BMI in the raw data was carried out using a robust linear regression method (Additional file 1: Figure S3B). Then, the obtained imputation data was randomly stratified into two parts (i.e., training and validation cohorts) under a ratio of 7:3.

### Feature selection

We adopted a rigorous approach for variable selection to identify the most relevant predictors for building the prediction model using the training cohort to avoid data leakage. Initially, a pairwise Pearson correlation matrix was employed to assess the clinical variables for collinearity, establishing a pairwise correlation threshold of r > 0.8. Collinearity arises when two or more predictor variables exhibit strong correlation, thereby complicating the assessment of each variable's distinct contribution to the outcome. So, we selected the most readily available variables among the collinear variables for further analysis. Subsequently, we utilized both the Boruta algorithm [13] and the Least Absolute Shrinkage and Selection Operator (LASSO) algorithm [14] in a two-step process.

The Boruta algorithm, a feature selection method based on random forests, iteratively assesses the importance of each variable by comparing it to the importance of randomly permuted versions of the same variable, allowing for the identification of truly relevant predictors by eliminating variables with importance levels comparable to random noise [13]. After applying the Boruta algorithm, a set of significant predictors was obtained.

Next, the LASSO algorithm was employed for further variable selection. LASSO is a regularization technique that performs both variable selection and coefficient estimation by imposing a constraint on the sum of the absolute values of the model parameters. This process results in some coefficients being shrunk to zero, effectively excluding them from the final model [15]. By using the LASSO algorithm, we obtained another set of significant predictors.

Finally, the intersection of the predictors identified by both the Boruta and LASSO algorithms was taken to ensure the inclusion of only the most relevant and robust variables in the development of our prediction model. This combined approach aimed to increase the model's accuracy and generalizability while reducing the risk of overfitting or including irrelevant predictors.

### Model development and validation

We utilized three machine learning classifiers—extreme gradient boosting (XGBoost), random forest (RF), and neural network (NN)—to construct predictive models for the risk of death within 72 h in patients with multiple traumas after admission. These algorithms have been explained elsewhere in detail. A brief summary is presented here. XGBoost, a prevalent and potent ensemble technique, is grounded in the gradient boosting framework and amalgamates predictions from multiple weak learners, predominantly decision trees, to generate a more accurate and robust model [16]. Similarly, RF algorithms employ tree-based models, aggregating numerous distinct decision trees through bootstrapping to enhance accuracy [17]. The NN, inspired by the general framework of neurons and neuronal circuitry, facilitates the passage of information from input nodes to hidden layers, optimizing the weights and mapping between input and output layers [18].

To ensure consistency, each model incorporated identical input variables. Subsequently, grid and random hyperparameter searches were employed to ascertain optimal hyperparameters for each model within the training data, utilizing the area under the receiver operating characteristic curve (AUROC) as the optimization metric. Following this process, model performance evaluation encompassed the area under the precision-recall curve (AUPRC), AUROC, calibration curve, Brier score, and Log Loss, while accuracy, sensitivity, specificity, positive predictive value (PPV), and negative predictive value (NPV) were calculated for a comprehensive assessment. Complementing the above metrics, decision curve analysis (DCA) [19] was conducted to quantify the net benefit at different threshold probabilities, evaluating the models' utility in decision-making. Finally, the Shapley Additive exPlanations (SHAP) algorithm [20] and Local Interpretable Model-Agnostic Explanations (LIME) [21] facilitated the provision of consistent and locally accurate values for each variable within the best-performing prediction model, further enhancing our understanding of the models' performance.

### Sample size calculation

In order to circumvent overfitting and secure enhanced precision in prognostic models, a sufficient sample size is imperative for the construction of predictive frameworks. We use a sample size calculated as $n={\left(\frac{1.96}{\delta }\right)}^{2}\phi \left(1-\phi \right)$, $\phi$ is the expected outcome ratio ($\phi$ = 0.29), $\delta$ is the set margin of error ($\delta$ = 0.05) [22]. As dictated by this formula, the minimal sample capacity for the training set employed in the model’s development amounts to 316 participants. The training population is obviously sufficient for model development.

According to Collins's recommendation for external validation of a prognostic model, a minimum of 100 events is required, ideally 200 or more [23]. The internal and external validation cohorts, with 206 and 200 events respectively, meet this standard.

### Statistical analysis

Continuous data were examined for normality, which was assessed using the Shapiro–Wilk test and presented as mean and standard deviation (SD) or median with interquartile range (IQR), as appropriate. The homogeneity of variance in groups was determined using the Levene test. If a Gaussian model of sampling was satisfied, parametric tests (unpaired two-tailed Student’s t-test or Welch's t-test for two groups, or one-way ANOVA for more than two groups) were used. Otherwise, non-parametric tests were used (Mann–Whitney U test for two groups, or Kruskal–Wallis H test for more than two groups). Categorical data were expressed as counts and percentages and compared using either the Chi-squared test or Fisher's exact test based on sample size and expected frequency in any cell. A two-sided *P* value less than 0.05 was considered statistically significant. All analyses were done with the R software, version 4.1.0.

## Results

### Patient characteristics

The Dryad data set encompassed 3075 adult polytrauma patients. To compensate for absent data, KNN imputation was employed for BMI in 1501 (48.8%), GCS in 43 (1.4%), pH in 832 (27.1%), BE in 703 (22.9%), and lactate in 472 (15.3%) (Additional file 1: Figure S2). The median patient age was 43 (IQR, 28–61) years. A total of 2363 (73.6%) patients were male, and 687 (22.3%) succumbed within 72 h following admission.

For patients from our intuition, the median patient age was 42 (IQR, 28–58) years. A total of 1232 (73.6%) patients were male, and 200 (12.0%) succumbed within 72 h following admission.

Of all the included individuals, 2153 were allocated to the training group, 922 to the internal validation group, and 1673 to the external validation group. Baseline characteristic distributions exhibited similarity across the three cohorts, except for GCS, pH, BE, and lactate (*P* < 0.05) (Table 1). Mortality rates were 481 (22.3%), 206 (22.3%), and 200 (12.0%) patients within the respective groups.

In the Dryad data set, compared to survivors, those who died showed a higher rate of age (42 [IQR, 27–58] vs. 51 [IQR, 32–73], *P* < 0.001), BMI (24.5 [IQR, 23.1–26.0] vs. 24.9 [IQR, 23.9–26.1], *P* < 0.001), ISS (26 [IQR, 20–34] vs. 34 [IQR, 25–50], *P* < 0.001), and lactate (2.10 [IQR, 1.40–3.02] vs. 3.30 [IQR, 2.20–5.61], *P* < 0.001), and presented a lower value in GCS (12 [IQR, 3–15] vs. 3 [IQR, 3–3], *P* < 0.001), pH (7.35 [IQR, 7.30–7.38] vs. 7.28 [IQR, 7.20–7.35], *P* < 0.001), and BE (− 2.50 [IQR, − 4.40–0.90] vs. − 5.30 [IQR, − 9.07–− 2.40], *P* < 0.001). No statistical difference was detected in gender between the two cohorts (*P* = 0.084) (Table 2). Similar phenomena were observed in the dataset derived from our institution (Additional file 1: Table S1).

**Table 2:** Baseline characteristics of patients from the Dryad database who died or survived within 72 h

	Alive (N = 2388)	Dead (N = 687)	*P* Value^c^
Demographic characteristics			
Age^a^, years	42 (27, 58)	51 (32, 73)	** < 0.001**
Sex^b^			0.084
Female	613 (25.7)	199 (29.0)	
Male	1775 (74.3)	488 (71.0)	
BMI^a^, kg/m^2	24.5 (23.1, 26.0)	24.9 (23.9, 26.1)	** < 0.001**
Scoring system			
ISS^a^	26 (20, 34)	34 (25, 50)	** < 0.001**
GCS^a^	12 (3, 15)	3 (3, 3)	** < 0.001**
Laboratory test			
pH^a^	7.35 (7.30, 7.38)	7.28 (7.20, 7.35)	** < 0.001**
BE^a^, mmol/L	− 2.50 (− 4.40, − 0.90)	− 5.30 (− 9.07, − 2.40)	** < 0.001**
Lactate^a^, mmol/L	2.10 (1.40, 3.02)	3.30 (2.20, 5.61)	** < 0.001**

Bold values indicate a *p*-value of less than 0.05

*BMI* body mass index, *ISS* injury severity score, *GCS* glasgow coma scale, *BE* base excess

^a^Values are presented as the median (inter-quartile range)

^b^Values are presented as number (percentage)

^c^*P* values between groups were assessed by the Chi-square and Mann–Whitney U tests

### Feature selection

**Fig. 2:**
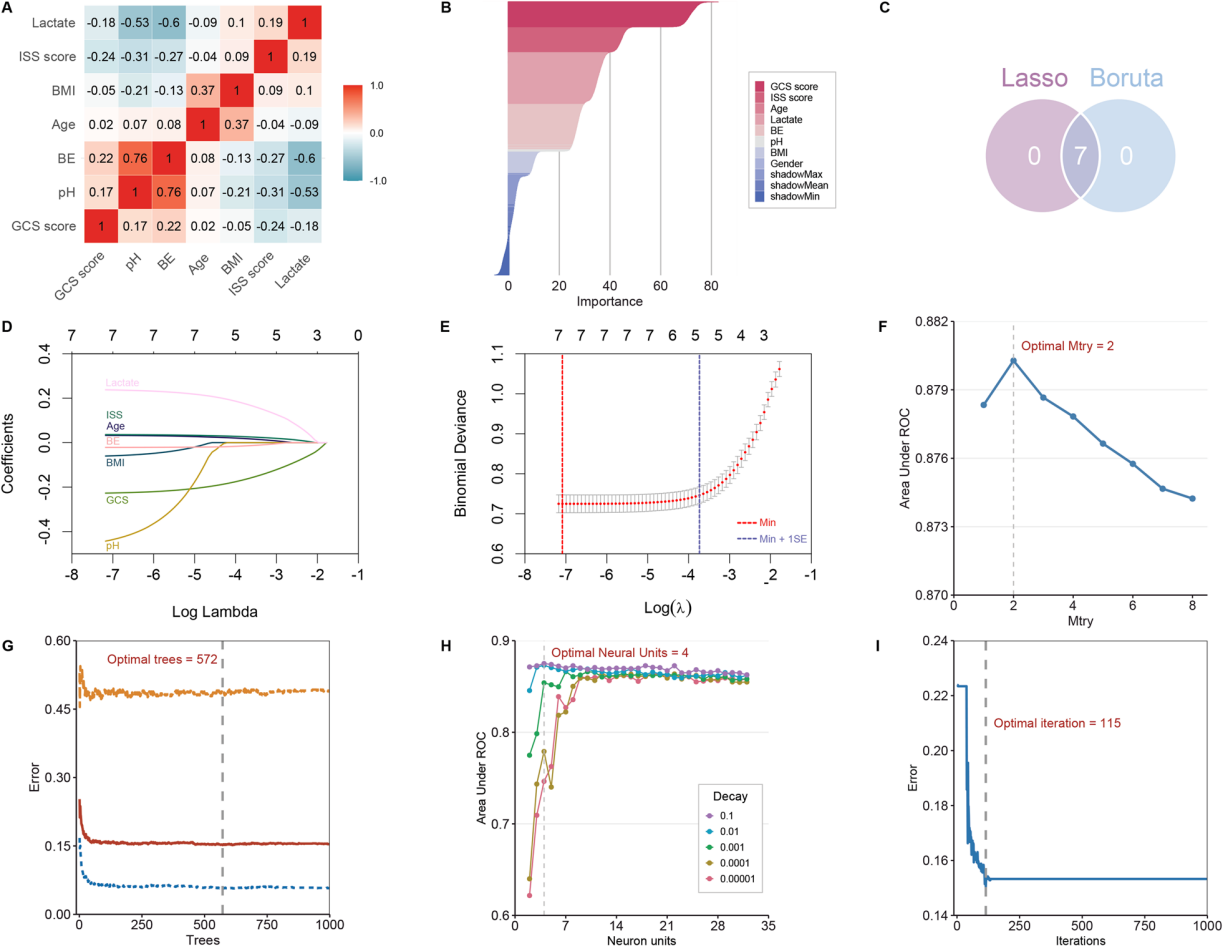
Selection of variables and model hyperparameters. **A** Spearman or Pearson correlation matrix of continuous clinical variables. “ × ” means that the *P* value is less than 0.05, which is not significant. **B** Variable selection by using the Boruta algorithm **C** Seven variables were determined by the Boruta and Lasso algorithms. **D**–**E** Variable selection by using the Lasso regression. **F**–**G** Determination of optimal hyperparameters for the random forest model. **H**–**I** Determination of optimal hyperparameters for the neural network model. *GCS* glasgow coma scale, *BE* base excess, *BMI* body mass index, *ISS* injury severity score, *Lasso* least absolute shrinkage and selection operator

As depicted in Fig. 2, none of the pairwise Pearson correlation values for continuous variables exceeded 0.8, indicating the absence of collinear variables. Consequently, all variables proceeded to the subsequent feature selection phase. Utilizing the Boruta (Fig. 2B) and LASSO (Fig. 2D, E) algorithms, a cumulative total of seven features emerged as significant predictors of the outcome. These encompassed age, BMI, BE, lactate, pH, ISS, and GCS. The chosen features were integrated into three machine learning classifiers—RF, NN, and XGBoost—to cultivate predictive models.

### Hyperparameters tuning

Fig. 2F–I, Additional file 1: Figures S4, S5 illustrate the process of grid and random hyperparameter searching for RF, NN, and XGBoost algorithms. The optimal *mtry* and *trees* are 2, and 572 for RF models. The optimal *neural units*, *decay*, and *iterations* are 4, 0.1 and 115 for NN models. The optimal *nrounds*, *max_depth*, *eta*, *gamma*, *colsample_bytree*, *min_child_weight*, and *subsample* were 980, 7, 0.1, 1, 0.9, 5, and 0.9.

### Development and validation of prediction models

**Fig. 3:**
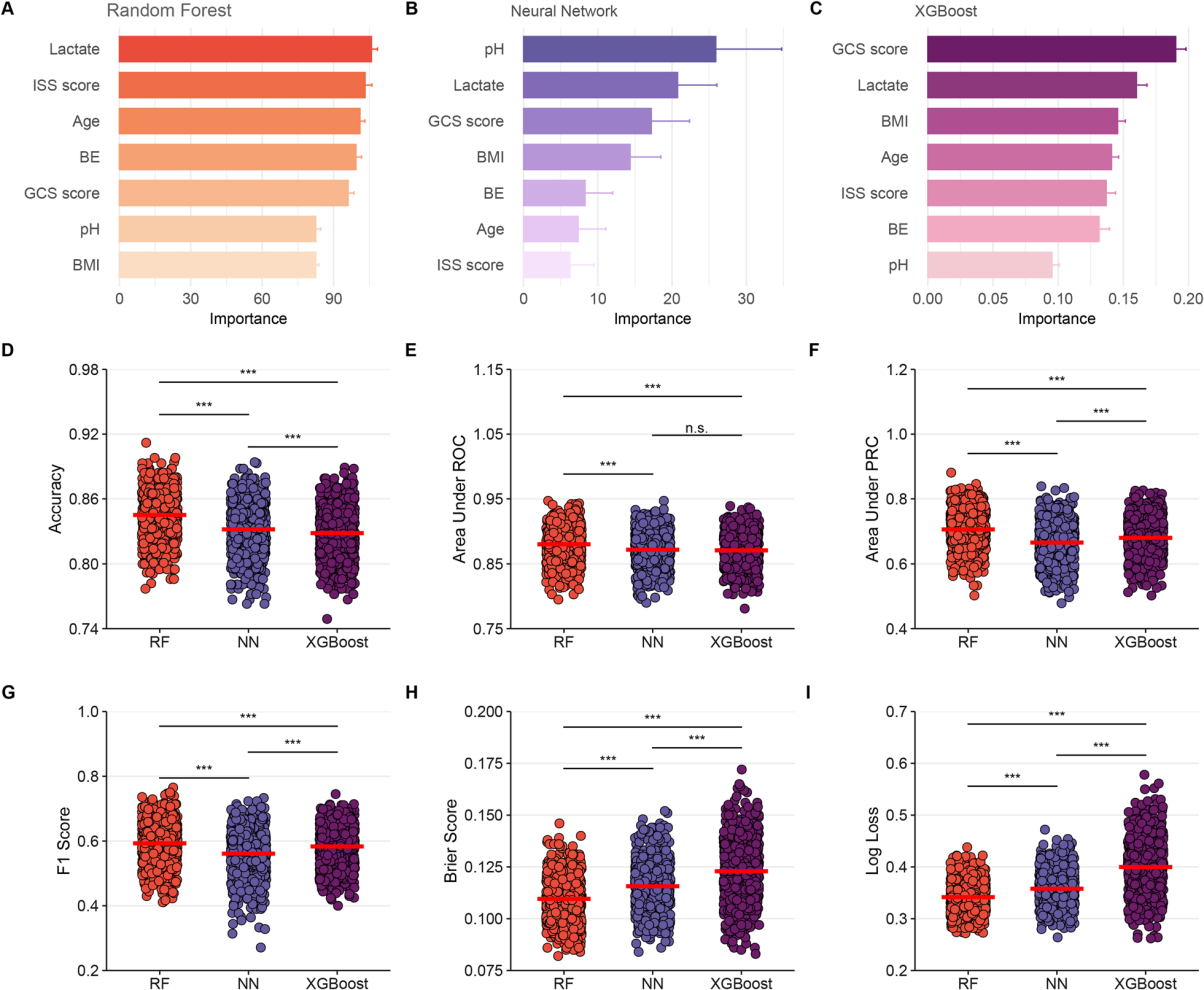
Variable importance and metrics for models with tenfold cross-validation repeated 100 times in development cohorts. ^***^P < 0.001. *ISS* injury severity score, *BE* base excess, *GCS* glasgow coma scale, *BMI* body mass index, *RF* random forest, *NN* neural network, *XGBoost* extreme gradient boosting, *ROC* receiver operating characteristic curve, *PRC* precision-recall curve

The above seven predictors and optimal hyperparameters were finally integrated into the 72 h death risk prediction models. The architecture of the NN is elucidated in Additional file 1: Figure S6. After tenfold cross-validation repeated 100 times in the training cohort, the mean and SD of the AUROCs for RF, NN, and XGBoost in predicting POST were 0.88 ± 0.02, 0.87 ± 0.02, and 0.87 ± 0.02, respectively (Additional file 1: Figures S7, S8, S9). The AUPRCs for RF, NN, and XGBoost were 0.65 ± 0.02, 0.63 ± 0.06, and 0.65 ± 0.06, respectively (Additional file 1: Figures S10, S11, S12). In RF, NN, and XGBoost, the calibration curves demonstrated good concordance between predicted and observed outcomes (Additional file 1: Figures S13, S14, S15). The Brier scores for RF, NN, and XGBoost were 0.11 ± 0.01, 0.12 ± 0.01, and 0.12 ± 0.01, respectively. The Log Losses for RF, NN, and XGBoost were 0.34 ± 0.03, 0.36 ± 0.03, and 0.40 ± 0.05, respectively. The accuracy for RF, NN, and XGBoost was 0.85 ± 0.02, 0.83 ± 0.02, and 0.83 ± 0.02, respectively. The F1 scores for RF, NN, and XGBoost were 0.59 ± 0.06, 0.56 ± 0.07, and 0.58 ± 0.06, respectively. The RF had the best performance in the internal validation for predicting the 72 h death risk (Fig. 3D–I).

**Fig. 4:**
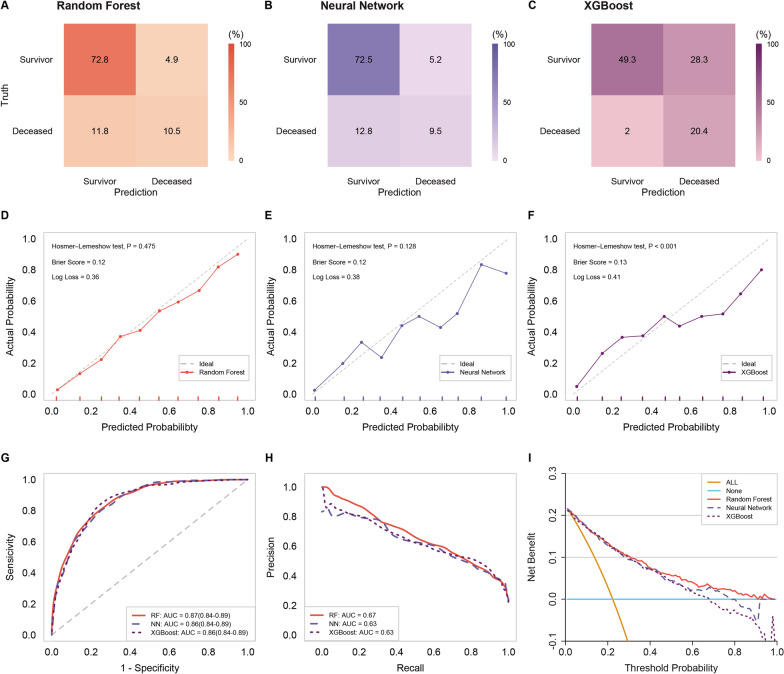
Confusion matrix plots, calibration plots, AUROCs, AUPRCs, and DCAs for models in validation cohorts. *RF* random forest, *NN* neural network, *XGBoost* extreme gradient boosting, *AUC* area under curve, *AUROC* area under the receiver operating characteristic curve, *AUPRC* area under the precision-recall curve, *DCA* decision curve analysis

In the internal validation cohort, the AUROCs for RF, NN, and XGBoost in predicting 72 h mortality risk after admission were 0.87 (95% confidence interval CI 0.84–0.89), 0.86 (95% CI 0.84–0.89), and 0.86 (95% CI 0.84–0.89), respectively. The AUPRCs for RF, NN, and XGBoost were 0.67 (95% CI 0.61–0.73), 0.63 (95% CI 0.55–0.70), and 0.63 (95% CI 0.56–0.71), respectively. Of the three machine learning models, the calibration curves demonstrated barely satisfactory concordance between predicted and observed outcomes, except for the XGBoost (*P* for Hosmer–Lemeshow test < 0.001). The Brier scores for RF, NN, and XGBoost were 0.12, 0.12, and 0.13, respectively. The Log Losses for RF, NN, and XGBoost were 0.36, 0.38, and 0.41, respectively (Fig. 4). Accuracy, sensitivity, specificity, PPV, NPV, and F1 score for RF were 0.83 (95% CI 0.81–0.86), 0.78 (95% CI 0.72–0.84), 0.78 (95% CI 0.75–0.81), 0.51 (95% CI 0.46–0.60), 0.93 (95% CI 0.90–0.94), and 0.56 (95% CI 0.52–0.60), respectively. NN had accuracy, sensitivity, specificity, PPV, NPV, and F1 score of 0.82 (95% CI 0.79–0.84), 0.80 (95% CI 0.74–0.85), 0.75 (95% CI 0.72–0.79), 0.49 (95% CI 0.44–0.58), 0.93 (95% CI 0.90–0.94), and 0.51 (95% CI 0.47–0.56). Accuracy, sensitivity, specificity, PPV, NPV, and F1 score for XGBoost were 0.70 (95% CI 0.67–0.73), 0.85 (95% CI 0.79–0.90), 0.75 (95% CI 0.72–0.78), 0.50 (95% CI 0.45–0.60), 0.95 (95% CI 0.92–0.95), and 0.57 (95% CI 0.52–0.61). As shown in Fig. 4 and Table 3, the RF also showed superior performance to the remaining models.

**Table 3:** Performance metrics for prediction models in the validation cohort

	Random Forest	Neural Network	XGBoost
Internal validation cohort			
Accuracy	**0.83 (0.81–0.86)**	0.82 (0.79–0.84)	0.70 (0.67–0.73)
Sensitivity	0.78 (0.72–0.84)	0.80 (0.74–0.85)	**0.85 (0.79–0.90)**
Specificity	**0.78 (0.75–0.81)**	0.75 (0.72–0.79)	0.75 (0.72–0.78)
PPV	**0.51 (0.46–0.60)**	0.49 (0.44–0.58)	0.50 (0.45–0.60)
NPV	0.93 (0.90–0.94)	0.93 (0.90–0.94)	**0.95 (0.92–0.95)**
F1 score	0.56 (0.52–0.60)	0.51 (0.47–0.56)	**0.57 (0.52–0.61)**
External validation cohort			
Accuracy	**0.97 (0.96–0.98)**	0.88 (0.86–0.89)	0.96 (0.94–0.96)
Sensitivity	**0.92 (0.87–0.95)**	0.82 (0.76–0.87)	**0.92 (0.87–0.95)**
Specificity	**0.95 (0.94–0.96)**	0.79 (0.77–0.81)	0.93 (0.92–0.95)
PPV	**0.71 (0.66–0.81)**	0.34 (0.32–0.44)	0.65 (0.61–0.77)
NPV	**0.99 (0.98–0.99)**	0.97 (0.96–0.97)	**0.99 (0.98–0.99)**
F1 score	**0.86 (0.82–0.90)**	0.43 (0.37–0.46)	0.81 (0.76–0.91)

Bold values indicate the best-performing model under the same evaluation criteria

95% confidence intervals are shown in parentheses

*PPV* positive predictive value; *NPV* negative predictive value

In the external validation cohort, the AUROCs for RF, NN, and XGBoost were 0.98 (95% CI 0.97–0.99), 0.87 (95% CI 0.85–0.89), and 0.96 (95% CI 0.95–0.98), respectively (Additional file 1: Figure S20G). The AUPRCs were 0.88 (95% CI 0.83–0.93), 0.47 (95% CI 0.41–0.54), and 0.81 (95% CI 0.74–0.87), respectively (Additional file 1: Figure S20H), with Brier scores of 0.03, 0.08, and 0.04, respectively. Log Losses were 0.14, 0.26, and 0.14, respectively. Calibration curves suggest that RF and XGBoost have better calibration than NN (Additional file 1: Figures S20D–F). Moreover, as depicted in Figures S20A to S20C and Table 3, RF consistently outperformed the other two models in terms of accuracy, sensitivity, specificity, PPV, NPV, and F1 score.

### Clinical usage of the models

In the training cohort, the DCA revealed that when the threshold probability exceeds 30%, the mean net benefits of RF for predicting polytrauma status after admission were superior to those of NN, XGBoost, and the strategies of treating all or none of the patients (Additional file 1: Figures S16, S17, S18). Similarly, in the validation cohorts, RF again demonstrated higher net benefits. Specifically, in the internal validation cohort, the advantage was seen when the threshold probability was over 27%, and in the external validation cohort, it was within the range of 36% to 66% (Fig. 4 and Additional file 1: S20I).

### Feature importance

Permutation feature importance analysis revealed the key predictors of early polytrauma status following admission. The results varied across the three models. In the RF model, lactate was the most influential factor, followed by ISS score, age, BE, GCS score, pH, and BMI. In the NN model, pH was the top predictor, with lactate, GCS score, BMI, and others coming next. In the XGBoost model, GCS score emerged as the most crucial predictor, followed by lactate, BMI, age, and others. To synthesize the significance of these variables across all three models, a rank score concept was introduced. The most important variable in each model received a full score of 7, and the score decreased sequentially down to 1 for the least significant variable. As shown in Additional file 1: Figure S19, lactate achieved a rank score of 19, placing it at the top. This underscores lactate’s substantial predictive value for assessing 72 h mortality risk after admission in polytrauma patients. The other top variables were the GCS score, with a rank score of 15, and age, with a score of 11, highlighting their importance in predictive modeling for this clinical scenario.

### Model explainability

**Fig. 5:**
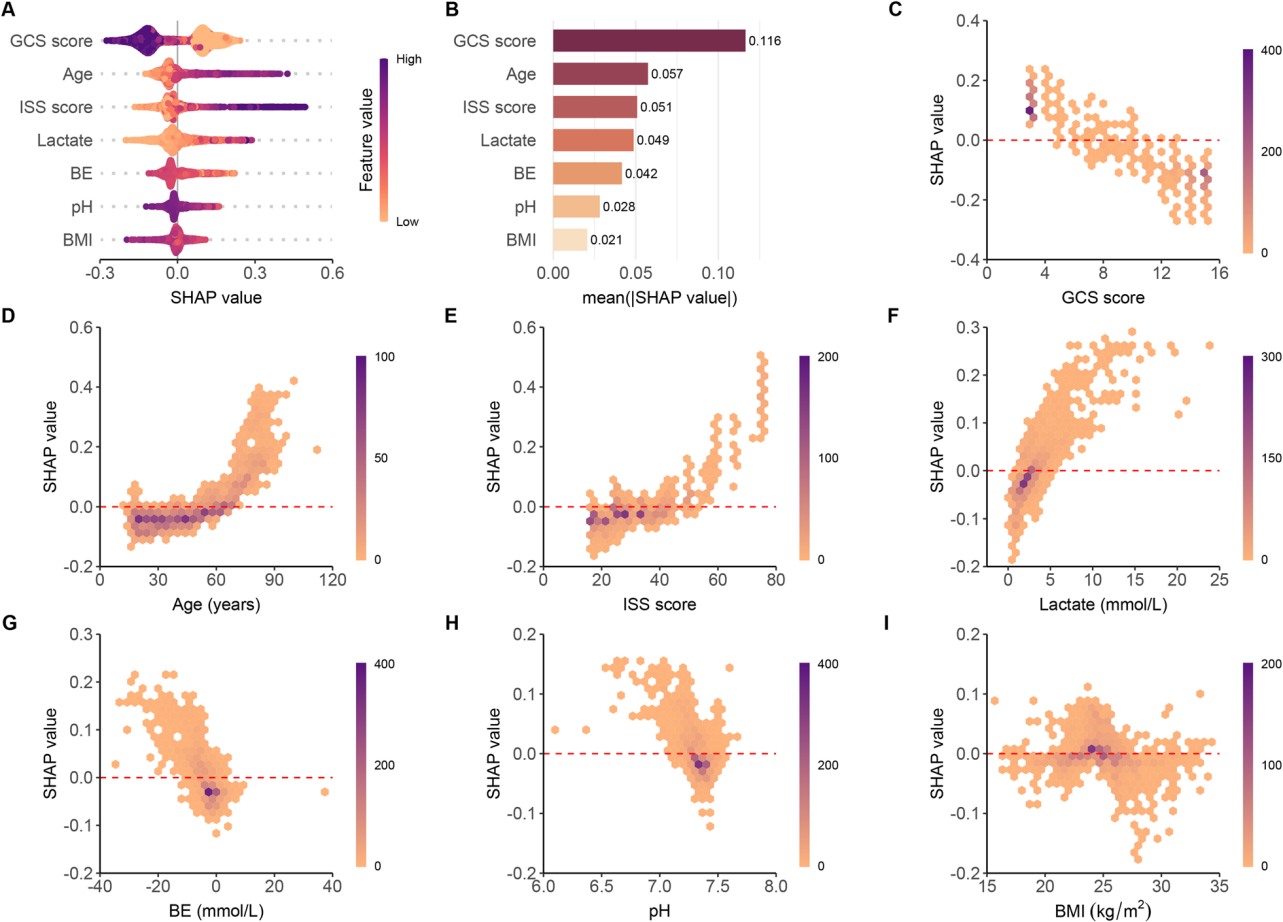
SHAP-based interpretation for the RF model. **A** The Beeswarm plot depicts the influence of the seven features across all model samples. Combining feature importance and feature effect, Beeswarm ranks the features according to the sum of the SHAP across all samples (y-axis). One row in the plot represents one feature, and each dot represents the feature Shapley value for one sample; colors represent feature values (purple for high, yellow for low). Long tails indicate that patient characteristics are of the utmost importance. The x-axis represents the influence on the model’s output, with positive values increasing risk and negative values decreasing risk. **B** Features are ranked according to the mean absolute Shapley values. **C**–**I** SHAP dependence plots show predicted risk versus feature value. *SHAP* shapley additive explanations, *RF* random forest, *GCS* glasgow coma scale, *ISS* injury severity score, *BE* base excess, *BMI* body mass index

The SHAP summary plot (Fig. 5A, B) and dependence plot (Fig. 5C–I) delineate the contributions of the seven predictors within the RF model. SHAP values exceeding zero signify an elevated risk of death within 72 h post-admission, while values below zero suggest a reduced risk. For instance, higher GCS scores (purple) generally yield SHAP values less than zero, indicating a diminished death risk in patients with elevated GCS scores. Moreover, Fig. 5B portrays the feature rankings based on the average absolute SHAP value. GCS score, age, ISS score, and lactate emerged as the four most influential variables in predictive power. Lower GCS scores, advanced age, heightened ISS scores, and increased lactate levels indicated a greater likelihood of death onset.

**Fig. 6:**
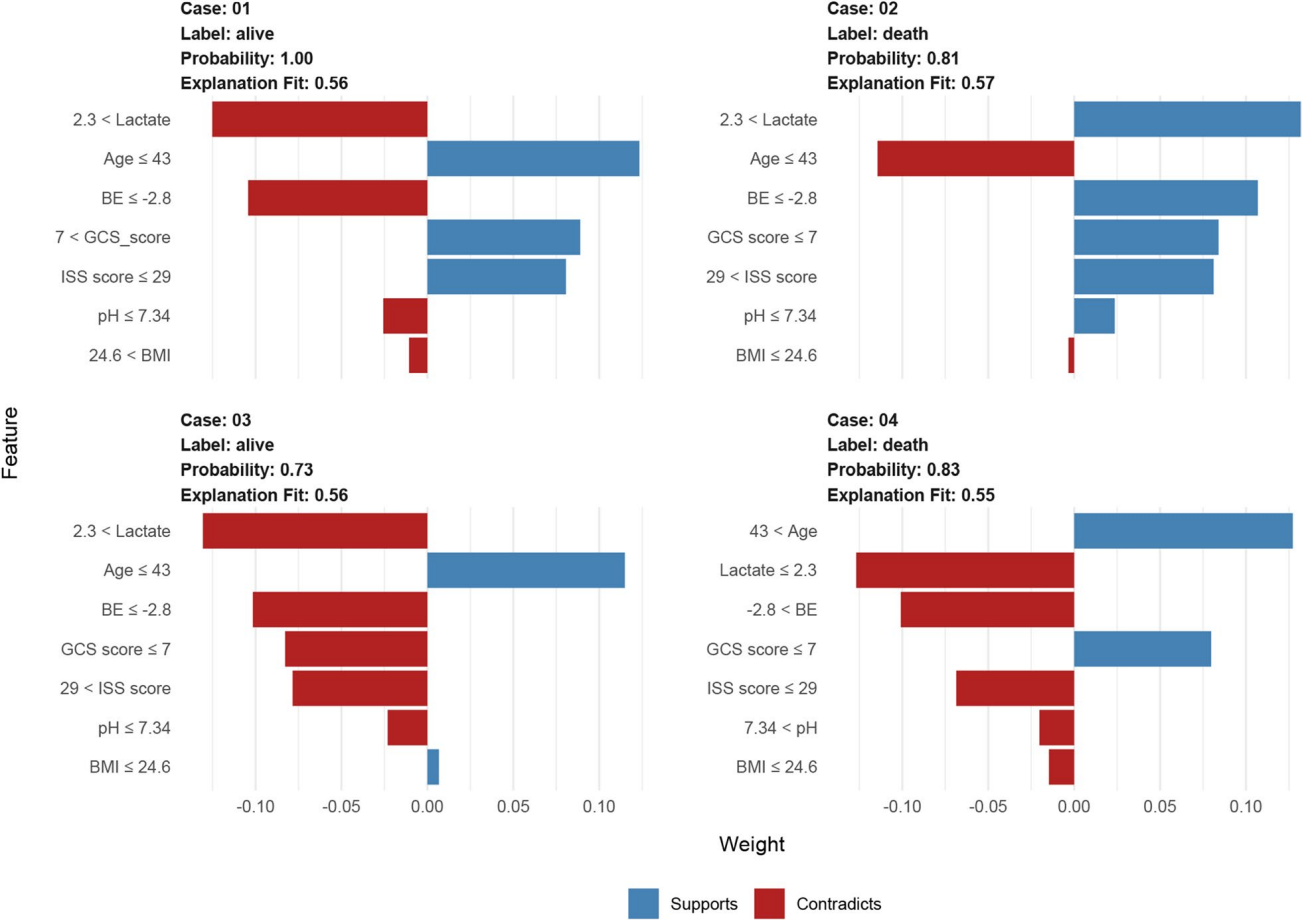
Interpretation of the RF with a local interpretable model explainer in four cases. Two living patients (cases 1 and 3) and two deceased patients (cases 2 and 4) are illustrated. Features with a blue bar favor the outcome, and those with a red bar contradict the outcome. The x-axis shows how much each feature adds or subtracts from the final probability value for the patient (i.e., a feature with a weight of 0.3 is equivalent to a 30% change in the probability of the outcome). *RF* random forest, *GCS* glasgow coma scale, *ISS* injury severity score, *BE* base excess, *BMI* body mass index

Then, the LIME explainer is applied to data generated by the RF model to explore the results of classification. The features for each case are presented in Fig. 6, with the weight of each feature represented in either blue or red depending on whether it favors the outcome or not. In case 1, the figure showed a confirmed probability of survival (100%), which may be attributed to the likelihood that the younger patient exhibiting an elevated GCS score and diminished ISS score possesses a greater probability of survival, despite the presence of adverse indicators, such as increased lactate and BMI levels, as well as decreased BE and pH values. In Case 2, the RF model forecasted a relatively elevated mortality probability of 81%. The interpreter algorithm discerned that a patient exhibiting heightened lactate, an increased ISS score, diminished BE, a reduced GCS score, and a lower pH might be predisposed to an unfavorable outcome, despite the presence of negative prognostic factors, such as a younger age and a lower BMI. It is noteworthy that despite the similarities between the indicators in Case 3 and Case 2, their outcomes diverge significantly. This discrepancy may be attributed to variations in the individual’s heterogeneity. In Case 4, the patient was assigned a high mortality risk probability of 83%. Favorable attributes, such as advanced age and a reduced GCS score, inclined the algorithm toward the outcome. Nevertheless, factors like lower lactate, diminished ISS score, low BMI, elevated BE, and higher pH constituted negative prognostic indicators for the outcome.

### Sensitivity analysis

To verify the robustness of the RF model, the seven variables used in building the model were divided into three categories: demographic characteristics (age and BMI), laboratory test variables (pH, BE, and lactate), and scoring variables (ISS and GCS). Three separate RF models were created for these distinct categories of variables, and their performance was compared in both internal and external validation sets, as shown in Additional file 1: Figures S21, S22. The comparison revealed that the comprehensive model, which incorporated all variables from the three categories, outperformed the others. Specifically, it performed better than the models solely based on scoring variables, laboratory test variables, or demographic variables. This result emphasizes the importance of considering a combination of different types of variables, including demographics, laboratory tests, and scoring metrics, to achieve the best predictive performance in modeling early polytrauma status after admission.

**Fig. 7:**
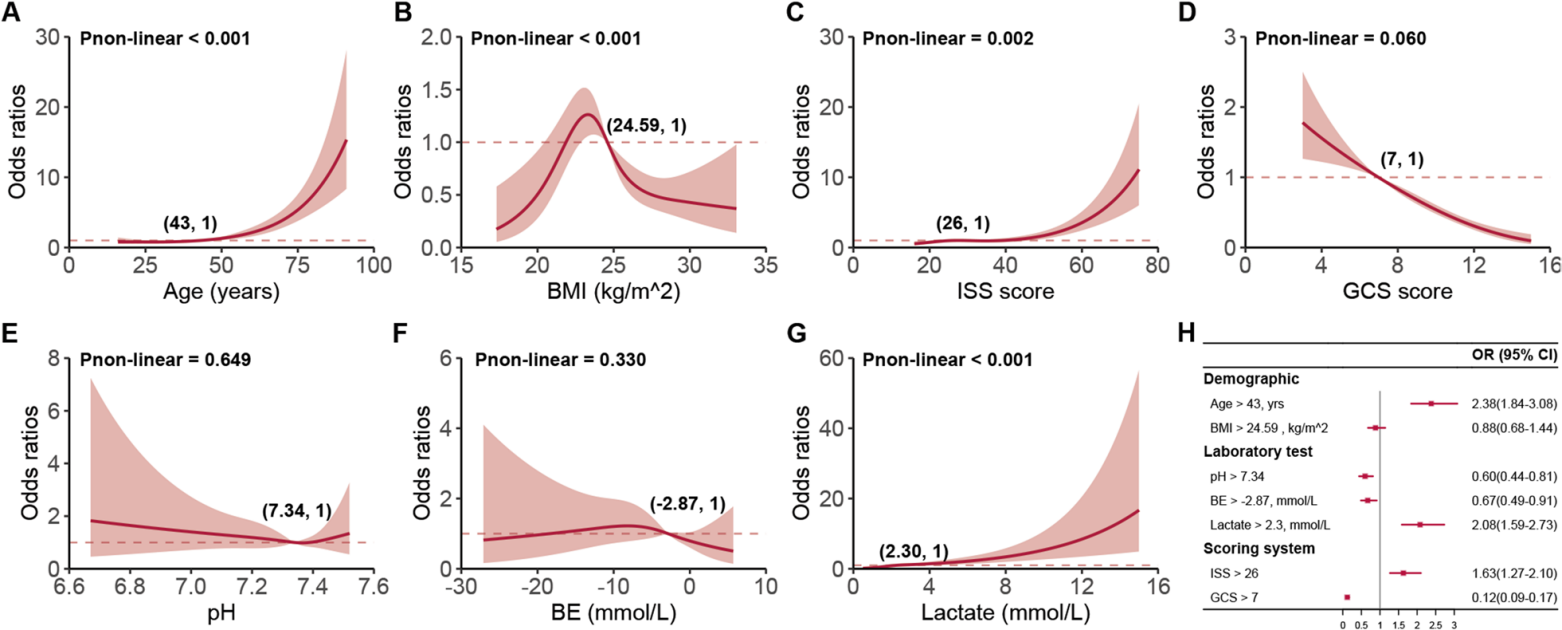
Restricted cubic spline analysis and forest plot. **A**–**G** Association between continuous predictors and 72 h mortality in polytrauma patients post-admission. For each curve, five knots at the 5th, 35th, 50th, 65th, and 95th percentiles were chosen. Solid lines denote odds ratios, while shaded regions represent 95% CIs. The cutoff point is the value nearest to or equal to the odds ratio at 1. **H** Adjusted odds ratios for categorical variables transformed from continuous ones based on cutoff points derived from the restricted cubic spline analysis. *BMI* body mass index, *GCS* galsgow coma scale, *ISS* injury severity score, *BE* base excess, *OR* odds ratio, *CI* confidence interval

The LIME explainer transforms the continuous predictors into categorical variables based on cutoff points derived from the quantile method. To test the accuracy of these cutoff values, a restricted cubic spline (RCS) analysis was used to identify an alternative set of cutoff points and categorize the seven continuous predictors once again. Following this transformation, a logistic regression model was built to investigate the associations between the predictors and the outcome. The LIME explainer and RCS analysis provided nearly identical cutoff points for age, BMI, ISS score, GCS score, pH, BE, and lactate (Figs. 6, 7). In the subsequent multivariable logistic regression analysis, deceased patients exhibited older age (odds ratio [OR], 2.38; 95% CI 1.84 to 3.08; *P* < 0.001), higher lactate (OR, 2.08; 95% CI 1.59 to 2.73; *P* < 0.001) and increased ISS score (OR, 1.63; 95% CI 1.59 to 2.73; *P* < 0.001), alongside lower pH (OR, 0.60; 95% CI 0.44 to 0.81; *P* < 0.001), a lower BE (OR, 0.67; 95% CI 0.49 to 0.91; *P* = 0.011), and lower GCS score (OR, 0.12; 95% CI 0.49 to 0.91; *P* < 0.001) (Fig. 7H). Remarkably, the findings from both the LIME interpretation and logistic regression were in agreement. Both methods identified age, lactate, and ISS score as factors that increase the likelihood of death within 72 h of admission, whereas pH, BE, and GCS score were found to decrease this likelihood. This consistency underscores the validity of the LIME explainer's transformations and highlights the key variables influencing mortality risk in patients with multiple injuries.

## Discussion

### Principal findings

Polytrauma is the leading cause of death among young and middle-aged individuals, and prompt, appropriate management of these injuries is essential for saving patients’ lives and facilitating their reintegration into society [24, 25]. Consequently, early risk stratification is particularly significant for patient management. In this study, we utilized machine learning techniques to develop and validate prediction models for 72 h mortality risk in adult polytrauma patients. By employing three distinct machine learning algorithms (RF, NN, and XGBoost), we identified seven critical predictors, including age, BMI, GCS score, ISS score, pH, BE, and lactate. The RF model demonstrated the best performance among the three models, both in terms of AUROC and other evaluation metrics, with good calibration and discriminative ability. Furthermore, decision curve analysis indicated that the RF model provided the highest net benefit in clinical settings when compared to the NN and XGBoost models.

Our importance analysis revealed that lactate levels were the most important predictor of 72 h mortality risk in polytrauma patients (Additional file 1: Figure S19), which is consistent with prior studies showing that lactate levels are a valuable prognostic indicator in trauma patients [24, 26]. Lactate levels reflect tissue hypoxia and the severity of the injury, and elevated levels are associated with increased morbidity and mortality [27, 28]. In a study encompassing 1,829 patients with blunt trauma, Gale et al. [29] verified that initial lactate levels were a reliable indicator of patients at an elevated risk of in-hospital mortality. In another observational cohort study involving 1075 trauma patients, Raux et al. [30] discovered that admission lactate was superior in predicting early fatalities, severe traumatic injuries, and extensive hemorrhaging. According to our investigation, patients with admission lactate levels exceeding the threshold of 2.30 mmol/L faced a 2.08-fold increased risk of death within 72 h compared to others (OR, 2.08; 95% CI 1.59 to 2.73) (Figs. 5A, F, 6, and 7H).

Admission BE is a recognized trauma marker that serves to assess injury severity and predict post-trauma outcomes [31]. Several studies [32] have indicated that an initial negative BE is associated with increased mortality risk in trauma patients. This relationship suggests that a lower BE corresponds to higher in-hospital mortality. This trend, which reveals a higher median BE in survivors compared to non-survivors, is consistently observed across different studies, and is also reflected in the data from our study. Specifically, the Dryad data showed values of − 2.50 (IQR − 4.40 to − 0.90) vs. − 5.30 (IQR, − 9.07 to − 2.40) and the external validation data had − 2.50 (IQR, − 4.30, − 0.90) vs. − 5.15 (IQR, − 8.55 to − 2.39), both of which were significant (*P* < 0.001) (Table 2 and Additional file 1: Table S1). Lichtveld et al. [33] concluded that BE was an independent predictor of mortality in patients with trauma, with an OR of 0.92 (95% CI 0.89–0.95), indicating an 8% increase in the risk of death for each unit reduction in BE. Our study produced similar findings, with an OR of 0.67 (95% CI 0.49 to 0.91), reinforcing the conclusion that lower BE is linked to a heightened risk of death (Figs. 5G, 6, and 7H). The slight differences in the ORs between the studies might be attributed to the varying populations enrolled in the respective studies. Despite these discrepancies, the collective evidence underscores the value of BE as a reliable and prognostic indicator for trauma patient outcomes, reinforcing its utility in clinical practice.

The association between pH and mortality in polytrauma patients is an important topic in emergency medicine and critical care [34]. Polytrauma refers to severe injuries sustained by a patient, involving multiple body regions or organ systems [34]. These injuries can result in a significant physiological stress response, leading to various complications and even death [35]. Blood pH is a measure of acidity or alkalinity, reflecting the balance of acids and bases in the body [36]. A normal blood pH range between 7.35 and 7.45 [37]. In our research, both the LIME algorithm and RCS analysis supported the finding that the optimal pH cutoff value is 7.34 (Figs. 6, 7H). In addition, according to the multivariable logistic regression, patients with a normal pH or higher have a lower mortality risk compared to those with a pH less than 7.34 (OR, 0.60; 95% CI 0.44 to 0.81) (Fig. 7H). This indicates that acidosis serves as an independent prognosticator of early mortality in patients with multiple traumas, as it compromises coagulation, diminishes cardiac contractility, and amplifies inflammation. In these patients, early identification and monitoring of blood pH disturbances is crucial for optimizing treatment strategies and reducing morbidity and mortality. Management strategies for acid–base disturbances include addressing the underlying cause, fluid resuscitation, blood transfusion, and, in some cases, the administration of buffering agents or mechanical ventilation adjustments. Maintaining a balance between correcting acid–base disturbances and avoiding overcorrection is important to minimize the risk of complications.

Age serves as another predictor of early mortality risk in patients with multiple traumas. In a meta-analysis of the elderly trauma population, Hashmi et al. [38] discovered that the risk of death increased with age and was twice as high in patients aged 74 compared to those aged 62. We concur with the findings of Hashmi and their colleagues. Moreover, we observed that the risk accelerated more rapidly when patients were over 50 years old (Fig. 7A). In comparison to those younger than 43, older individuals faced an additional 2.38-fold risk of death (OR, 2.38; 95% CI 1.84–3.08) (Figs. 5A, 6, 7H). This highlights the need for greater attention for both middle-aged and elderly patients with multiple traumas. The slight discrepancy between our results and those of Hashmi et al. could be attributed to diverse study populations and distinct statistical methods employed for analysis.

Other significant predictors in our study included the GCS score and the ISS score, which are well-established factors affecting trauma outcomes [39, 40]. The former, a crucial measure of neurological function and the severity of head injury, boasts advantages such as simplicity, practicality, time-efficiency, and cost-effectiveness [41]. Several authors have determined that a low GCS score is associated with poor outcomes, which is consistent with our evidence from the SHAP summary plot (Fig. 5A) and dependence plot (Fig. 5C), as well as the RCS analysis of GCS in Fig. 7D. In contrast to GCS, the probability of patient survival decreases with increasing ISS scores [42]. Watts et al. [43] reported that ISS scores were positively associated with in-hospital mortality in elderly trauma patients. This is not an isolated finding. In our study, compared to patients with ISS scores less than 25, the risk of death was approximately 1.63-fold higher (OR, 1.63; 95% CI 1.27–2.10) for those patients with larger values. However, the cutoff value of ISS was 29 in the LIME analysis. This discrepancy may be attributed to the limitations of the logistic regression approach, particularly its linearity assumption. Despite diligently building RCS models to explore this assumption, the residual complexity in the predictor-response variable relationship may still have been overlooked. Such challenges can often be addressed by machine learning algorithms, which do not necessitate strict data structure assumptions and possess the capability to learn complex functional forms through non-parametric methods [44]. In future studies, we will continue to investigate the correlation between the diagnostic efficacy of various ISS cutoff values and patient prognosis.

Based on these predictors, the RF model exhibited strong discrimination and calibration in both the training, internal and external validation cohorts, achieving an AUROC of 0.88, 0.87 and 0.98 respectively (Additional file 1: Figures S7, 4G and S20G). These outcomes indicate that the RF model can effectively predict the 72 h mortality risk in adult polytrauma patients, surpassing the performance of both the NN and XGBoost models. Furthermore, the RF model showcased superior clinical utility, as demonstrated by the higher net benefits in the decision curve analysis (Fig. 4I and Additional file 1: Figures S16, S20I). These insights emphasize the potential of the RF model to be employed in clinical settings to aid decision-making and allocate resources more effectively. In the sensitivity analysis, the RF model retained its standout performance when compared with other random forest models utilizing various demographic characteristics, laboratory test variables, and scoring variables (Additional file 1: Figures S21, S22). This further strengthens the notion that the variables selected for constructing our model were indeed optimal and the RF model could serve as a robust tool in trauma care.

## Strengths

Our study has several strengths, including a large sample size, a rigorous model development and validation process, and the use of multiple machine learning algorithms to identify the best-performing model. Additionally, we employed various evaluation metrics and model explainability techniques, such as SHAP and LIME, to ensure transparency and facilitate the interpretation of the results. Finally, we performed sensitivity analyses to test the robustness of our findings.

## Limitations

However, there are also limitations to our study. First, the assumption that the Dryad data was missing completely at random, along with our use of KNN imputation to deal with missing data, could accidentally introduce bias if these assumptions are broken. In addition, the retrospective design and partial data unavailability of this study might have led to the exclusion of potentially relevant predictors such as APACHE II and SOFA scores. This may limit the model's ability to capture all the nuances of the problem, potentially reducing its predictive accuracy and generalizability. Thirdly, the dataset used to build the models spans from 1993 to 2013. Medical practices and standards can change over time, and the dataset might not represent current patient populations or medical techniques. Although the models were validated with more recent data from the institution and showed robust performance, there might still be concerns about the applicability of the models to different populations or changing clinical practices. Furthermore, while the model demonstrated good performance within the institution, there might be questions about how well the model would generalize to other healthcare settings. It would be crucial to further validate the model in diverse populations to ensure its broader applicability.

## Conclusions

In conclusion, we developed and validated an RF model for predicting 72 h mortality risk in adult polytrauma patients using machine learning techniques. The model demonstrated good discrimination and calibration, as well as superior clinical utility when compared to NN and XGBoost models. The identified predictors, such as lactate, GCS score, and age, could guide clinical decision-making and resource allocation in the management of polytrauma patients. Future studies should focus on validating and refining the model in different settings and populations, as well as exploring the potential integration of other relevant predictors to improve the model's performance.

### Supplementary Information

**Additional file 1: Table S1.** Baseline characteristics of patients from external cohort who died or survived within 72 h. **Figure S1.** Cohort and sample selection.This flow diagram shows patient inclusion and exclusion criteria in each cohort as well as the dataset partition for training, internal and external validation cohorts. **Figure S2.** Proportion of missings for each variable. **Figure S3.** Comparisons between raw data and imputation data. (A), (C)-(F) Missing data of each variable was imputed by using the KNN algorithm. (B)Missing data was imputed by combining the KNN algorithm and robust multivariable linear regression. **Figure S4. ** Grid search method to determine hyperparameters of XGBoost models. **Figure S5.** Grid search method to determine best rounds of XGBoost models. **Figure S6.** Neural network interpretation diagram. **Figure S7.** ROCs for RF models in training dataset with 10-fold internal cross-validation repeated 100 times. **Figure S8.** ROCs for NN models in training dataset with 10-fold internal cross-validation repeated 100 times. **Figure S9.** ROCs for XGBoost models in training dataset with 10-fold internal cross-validation repeated 100 times. **Figure S10.** PRs for RF models in training dataset with 10-fold internal cross-validation repeated 100 times. **Figure S11.** PRs for NN models in training dataset with 10-fold internal cross-validation repeated 100 times. **Figure S12.** PRs for XGBoostmodels in training dataset with 10-fold internal cross-validation repeated 100 times. **Figure S13.** Calibrate plots for RF models in training dataset with 10-fold internal cross-validation repeated 100 times. **Figure S14.** Calibrate plots for NNmodels in training dataset with 10-fold internal cross-validation repeated 100 times. **Figure S15.** Calibrate plots for XGBoost models in training dataset with 10-fold internal cross-validation repeated 100 times. **Figure S16.** Decision curves for RF models in training dataset with 10-fold internal cross-validation repeated 100 times. **Figure S17.** Decision curves for NN models in training dataset with 10-fold internal cross-validation repeated 100 times. **Figure S18.** Decision curves for XGBoost models in training dataset with 10-fold internal cross-validation repeated 100 times. **Figure S19.** Rank score of importance for predictors. **Figure S20.** Confusion matrix plots, calibration plots, AUROCs, AUPRCs, and DCAs for models in the external validation cohorts. **Figure S21.** AUROCs, AUPRCs, calibration plots, and DCA for random forest models in the internal validation cohorts. **Figure S22.** AUROCs, AUPRCs, calibration plots, and DCA for random forest models in the external validation cohorts.

## Data Availability

The data for model training and internal validation in the current study is openly available in the Dryad database [10]. The external validation data for this study are available on request to the corresponding author.

## Abbreviations

AUC: Area under curve
AUPRC: Area under the precision-recall curve
AUROC: Area under the receiver operating characteristic curve
BE: Base excess
BMI: Body mass index
DCA: Decision curve analysis
GCS: Glasgow coma scale
ISS: Injury severity score
Lasso: Least absolute shrinkage and selection operator
LIME: Local interpretable model-agnostic explanations
NN: Neural network
PRC: Precision-recall curve
RF: Random forest
ROC: Receiver operating characteristic curve
SHAP: Shapley additive explanations
XGBoost: Extreme gradient boosting
