# Supplementary material for: Advancing polytrauma care: developing and validating machine learning models for early mortality prediction
Source: J Transl Med. 2023 Sep 25;21:664. doi: 10.1186/s12967-023-04487-8 (PMC10518974; doi:10.1186/s12967-023-04487-8)
Supplement: Supplementary file 1 — Additional file 1: Table S1. Baseline characteristics of patients from external cohort who died or survived within 72 h. Figure S1. Cohort and sample selection.This flow diagram shows patient inclusion and exclusion criteria in each cohort as well as the dataset partition for training, internal and external validation cohorts. Figure S2. Proportion of missings for each variable. Figure S3. Comparisons between raw data and imputation data. (A), (C)-(F) Missing data of each variable was imputed by using the KNN algorithm. (B)Missing data was imputed by combining the KNN algorithm and robust multivariable linear regression. Figure S4. Grid search method to determine hyperparameters of XGBoost models. Figure S5. Grid search method to determine best rounds of XGBoost models. Figure S6. Neural network interpretation diagram. Figure S7. ROCs for RF models in training dataset with 10-fold internal cross-validation repeated 100 times. Figure S8. ROCs for NN models in training dataset with 10-fold internal cross-validation repeated 100 times. Figure S9. ROCs for XGBoost models in training dataset with 10-fold internal cross-validation repeated 100 times. Figure S10. PRs for RF models in training dataset with 10-fold internal cross-validation repeated 100 times. Figure S11. PRs for NN models in training dataset with 10-fold internal cross-validation repeated 100 times. Figure S12. PRs for XGBoostmodels in training dataset with 10-fold internal cross-validation repeated 100 times. Figure S13. Calibrate plots for RF models in training dataset with 10-fold internal cross-validation repeated 100 times. Figure S14. Calibrate plots for NNmodels in training dataset with 10-fold internal cross-validation repeated 100 times. Figure S15. Calibrate plots for XGBoost models in training dataset with 10-fold internal cross-validation repeated 100 times. Figure S16. Decision curves for RF models in training dataset with 10-fold internal cross-validation repeated 100 times. Figure S17. Decisi [file 12967_2023_4487_MOESM1_ESM.pdf]

Advancing Polytrauma Care: Developing and Validating Machine Learning Models for Early Mortality Prediction

Additional Materials

Table of contents.

Item	Description	Page
Table S1	Baseline characteristics of patients from external cohort who died or survived within 72 hours.	3
Figure S1	Cohort and sample selection.	4
Figure S2	Proportion of missings for each variable.	5
Figure S3	Comparisons between raw data and imputation data.	6
Figure S4	Grid search method to determine hyperparameters of XGBoost models.	7
Figure S5	Grid search method to determine best rounds of XGBoost models.	8
Figure S6	Neural network interpretation diagram.	9
Figure S7	ROCs for RF models in training dataset with 10-fold internal cross-validation repeated 100 times.	10
Figure S8	ROCs for NN models in training dataset with 10-fold internal cross-validation repeated 100 times.	11
Figure S9	ROCs for XGBoost models in training dataset with 10-fold internal cross-validation repeated 100 times.	12
Figure S10	PRs for RF models in training dataset with 10-fold internal cross-validation repeated 100 times.	13
Figure S11	PRs for NN models in training dataset with 10-fold internal cross-validation repeated 100 times.	14
Figure S12	PRs for XGBoost models in training dataset with 10-fold internal cross-validation repeated 100 times.	15
Figure S13	Calibrate plots for RF models in training dataset with 10-fold internal cross-validation repeated 100 times.	16

Table of contents (continued).

Item	Description	Page
Figure S14	Calibrate plots for NN models in training dataset with 10-fold internal cross-validation repeated 100 times.	17
Figure S15	Calibrate plots for XGBoost models in training dataset with 10-fold internal cross-validation repeated 100 times.	18
Figure S16	Decision curves for RF models in training dataset with 10-fold internal cross-validation repeated 100 times.	19
Figure S17	Decision curves for NN models in training dataset with 10-fold internal cross-validation repeated 100 times.	20
Figure S18	Decision curves for XGBoost models in training dataset with 10-fold internal cross-validation repeated 100 times.	21
Figure S19	Rank score of importance for predictors.	22
Figure S20	Confusion matrix plots, calibration plots, AUROCs, AUPRCs, and DCAs for models in the external cohort.	23
Figure S21	AUROCs, AUPRCs, calibration plots, and DCA for random forest models in the internal cohort.	24
Figure S22	AUROCs, AUPRCs, calibration plots, and DCA for random forest models in the external cohort.	25

Table S1. Baseline characteristics of patients from external cohort who died or survived within 72 hours.

	Alive (N = 1473)	Dead (N = 200)	*P* Value*
Demographic characteristics			
Age†, years	41 (28, 56)	48 (32, 68)	**<0.001**
Sex¶			
female	385 (26.1)	56 (28.0)	0.575
male	1088 (73.9)	144 (72.0)	
BMI†, kg/m^2	24.5 (22.3, 26.3)	25.1 (23.0, 27.4)	**<0.001**
Scoring system			
ISS†	27 (21, 34)	29 (25, 43)	**<0.001**
GCS†	14 (3, 15)	3 (3, 3)	**<0.001**
Laboratory test			
pH†	7.35 (7.30, 7.38)	7.29 (7.19, 7.36)	**<0.001**
BE†, mmol/L	-2.50 (-4.30, -0.90)	-5.15 (-8.55, -2.39)	**<0.001**
Lactate†, mmol/L	2.10 (1.40, 3.00)	3.37 (2.30, 5.58)	**<0.001**

†Values are presented as the median (inter-quartile range).

¶Values are presented as number (percentage).

* *P* values between groups were assessed by the Chi-square and Mann-Whitney U tests.

Abbreviations: BMI, body mass index; ISS, injury severity score; GCS, glasgow coma scale; BE, base excess.

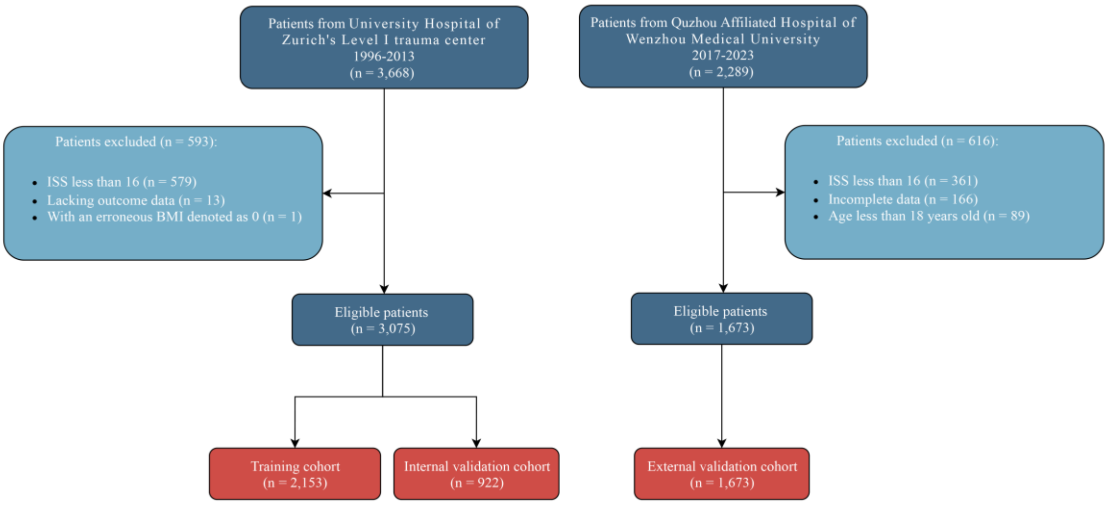

Figure S1. Cohort and sample selection. This flow diagram shows patient inclusion and exclusion criteria in each cohort as well as the dataset partition for training, internal and external validation cohorts.

Abbreviations: ISS, injury severity score.

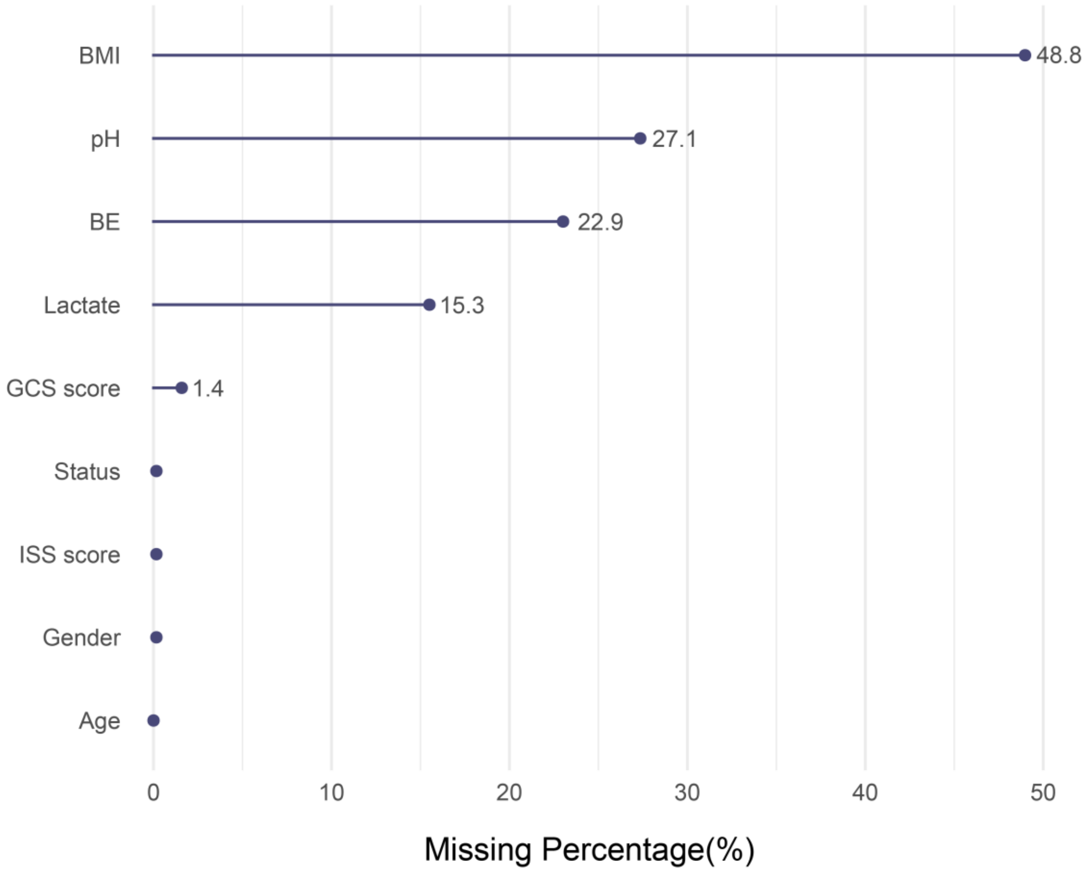

Figure S2. Proportion of missings for each variable.

Abbreviations: BMI, body mass index; BE, base excess; GCS, Glasgow coma scale; ISS, injury severity score.

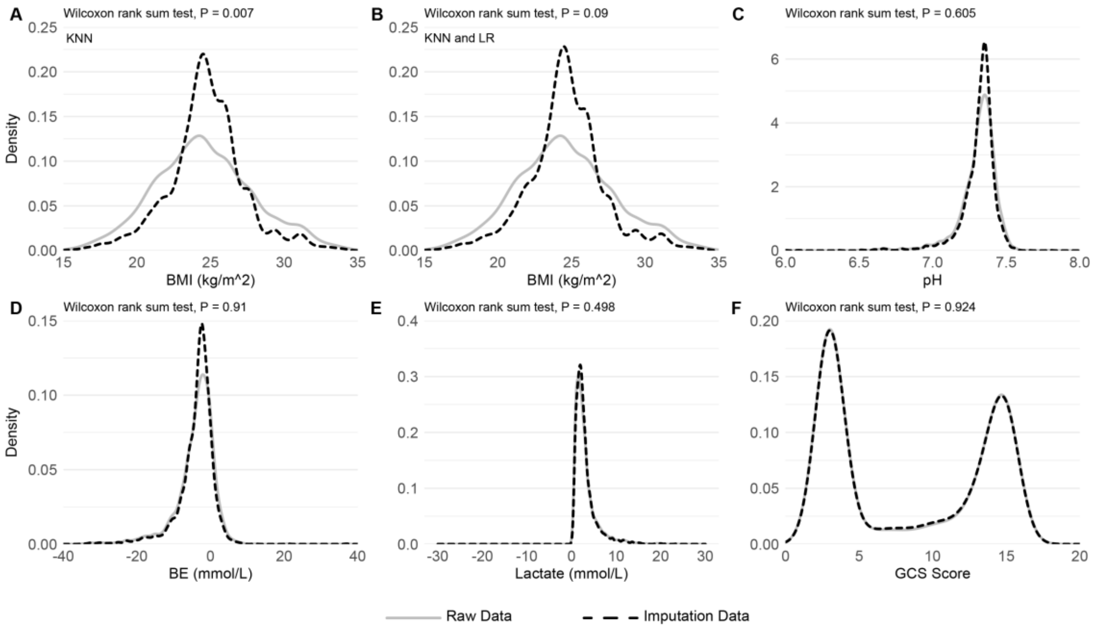

Figure S3. Comparisons between raw data and imputation data. (A), (C)-(F) Missing data of each variable was imputed by using the KNN algorithm. (B) Missing data was imputed by combining the KNN algorithm and robust multivariable linear regression.

Abbreviations: KNN, k-nearest neighbor; BMI, body mass index; BE, base excess; GCS, Glasgow coma scale; ISS, injury severity score.

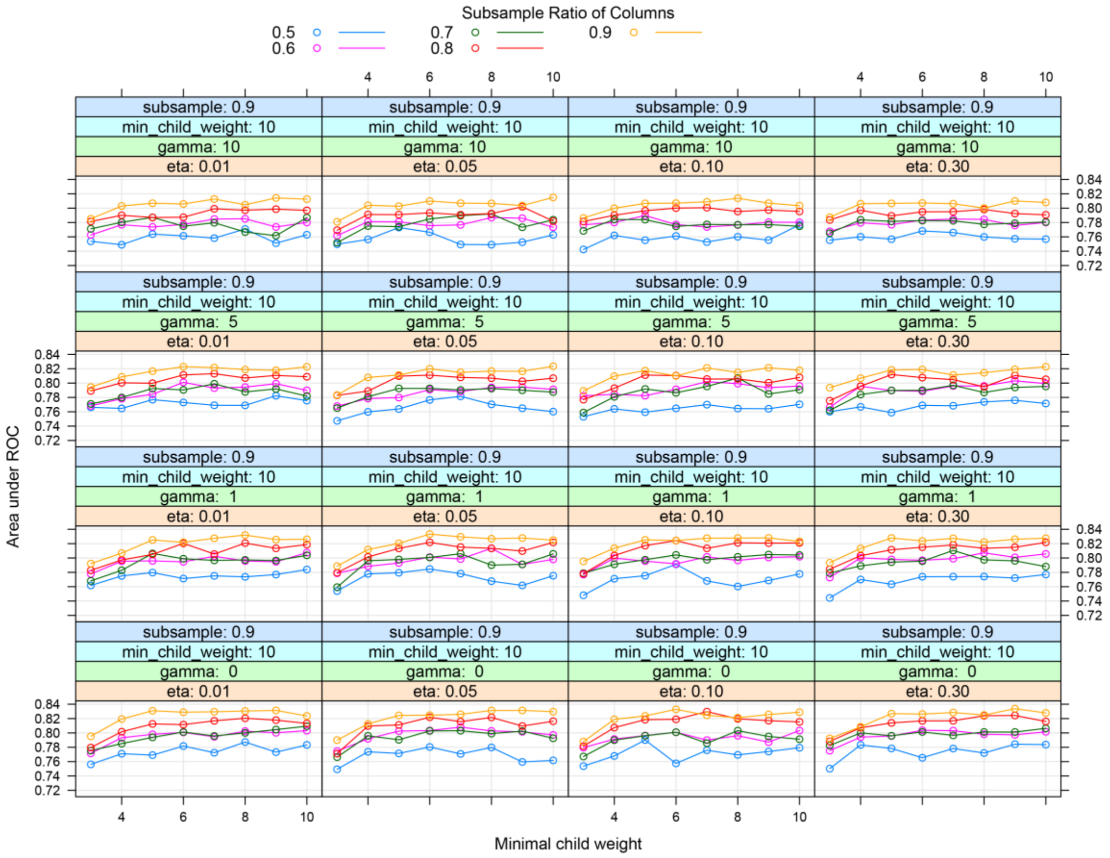

Figure S4. Grid search method to determine hyperparameters of XGBoost models.

Abbreviations: XGBoost, extreme gradient boosting; ROC, receiver operating characteristic curve.

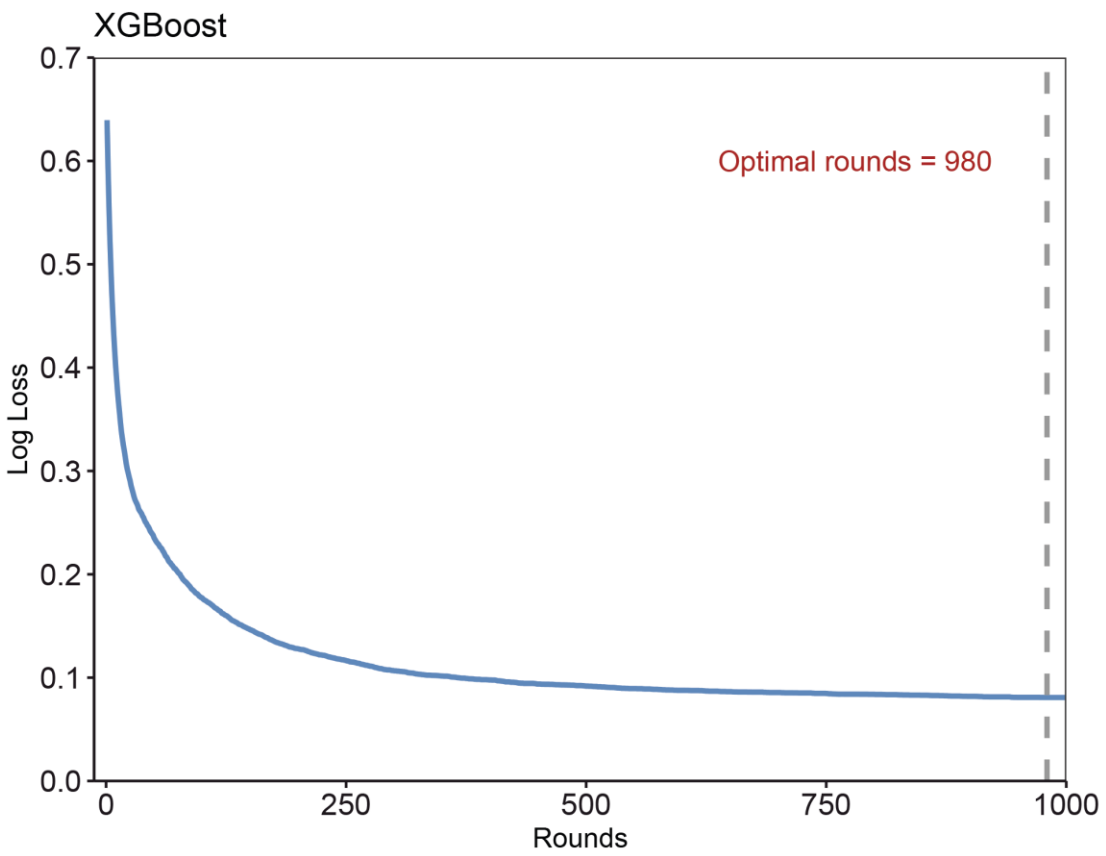

Figure S5. Grid search method to determine best rounds of XGBoost models.

Abbreviations: XGBoost, extreme gradient boosting.

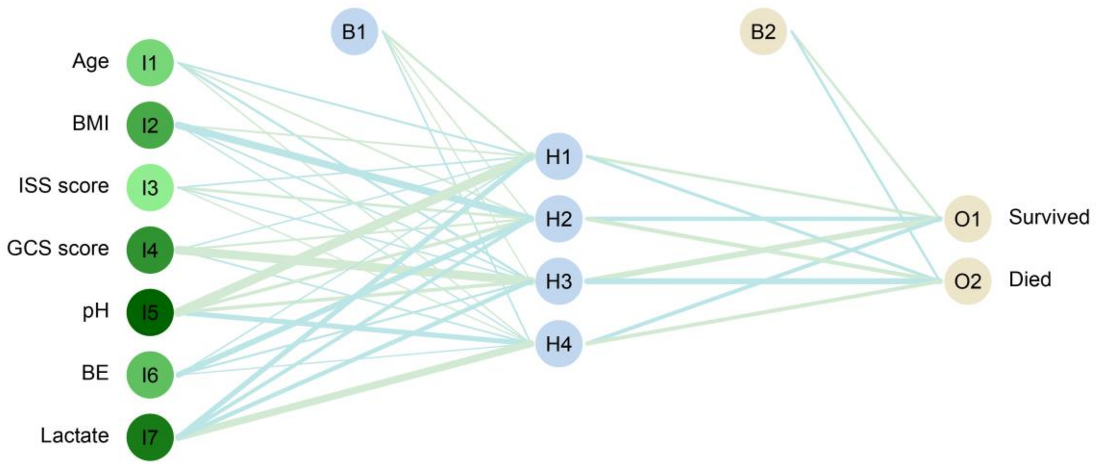

Figure S6. Neural network interpretation diagram. The light green lines indicate positive weights and light blue lines indicate negative weights. The first layer receives the input variables (I1 through I7) and each is connected to all nodes in the hidden layer (H1 through H4). The output layers (O1through O2) are connected to all hidden layer nodes. Bias nodes (B1 through B2) provide a function that is similar to the intercept term in a linear model and are shown as connections to the hidden and output layers in the plot.

Abbreviations: BMI, body mass index; ISS, injury severity score; GCS, Glasgow coma scale; BE, base excess.

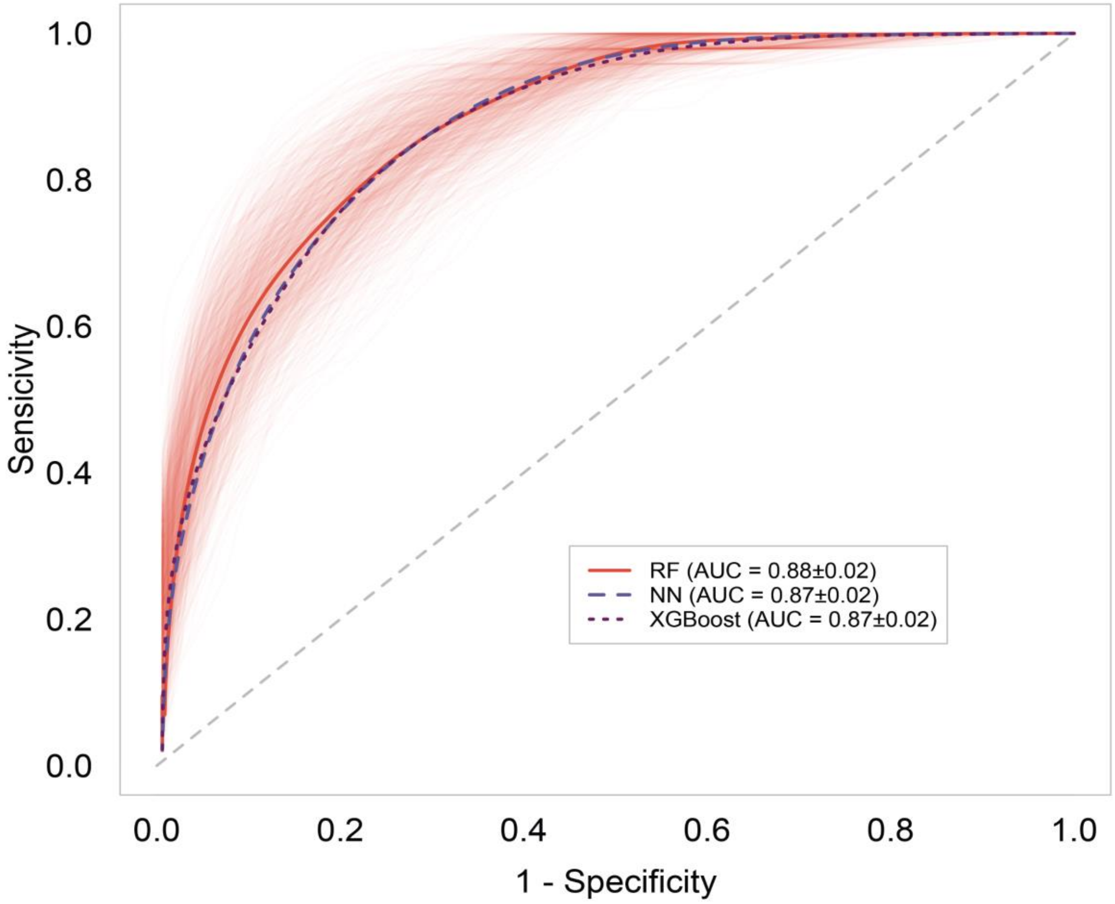

Figure S7. ROCs for RF models in training dataset with 10-fold internal cross-validation repeated 100 times.

Abbreviations: ROCs, receiver operating characteristic curves; RF, random forest; NN, neural network; XGBoost, extreme gradient boosting.

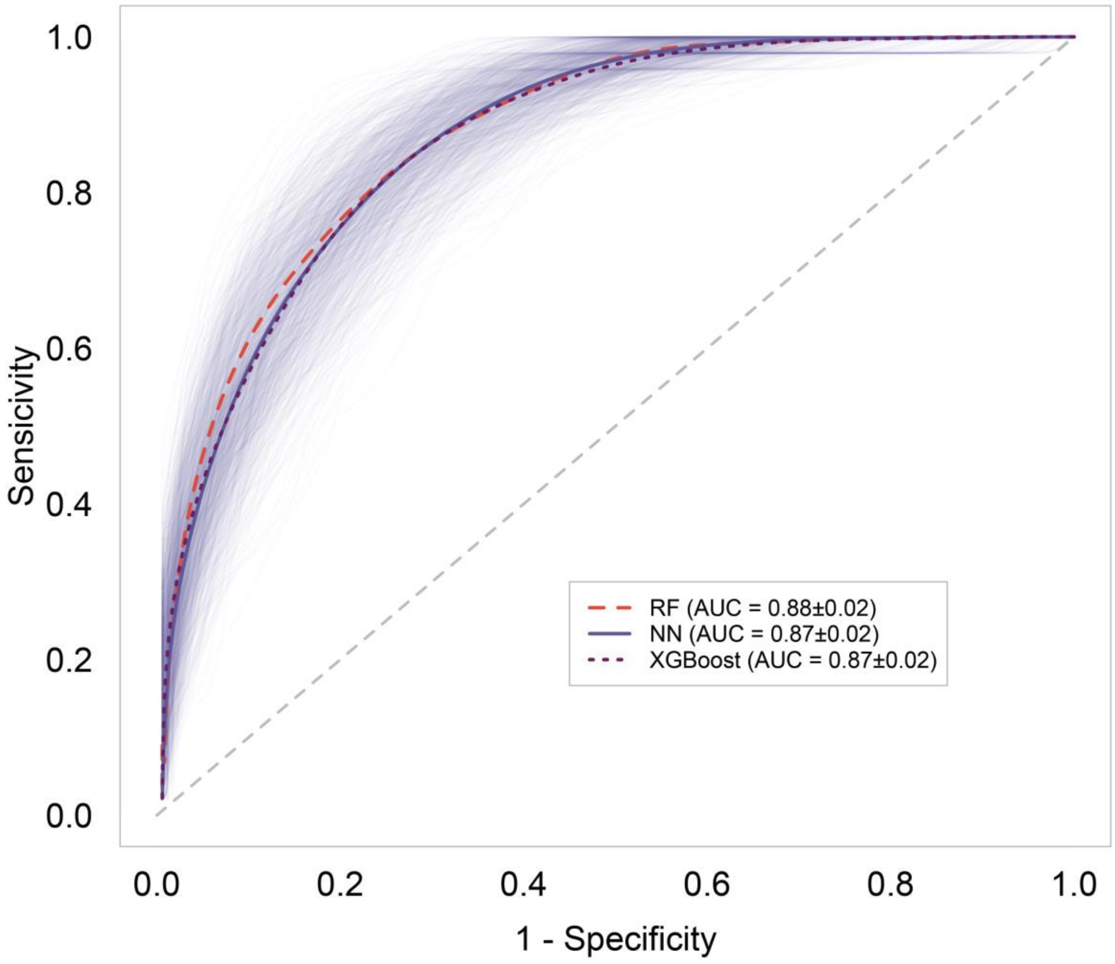

Figure S8. ROCs for NN models in training dataset with 10-fold internal cross-validation repeated 100 times.

Abbreviations: ROCs, receiver operating characteristic curves; RF, random forest; NN, neural network; XGBoost, extreme gradient boosting.

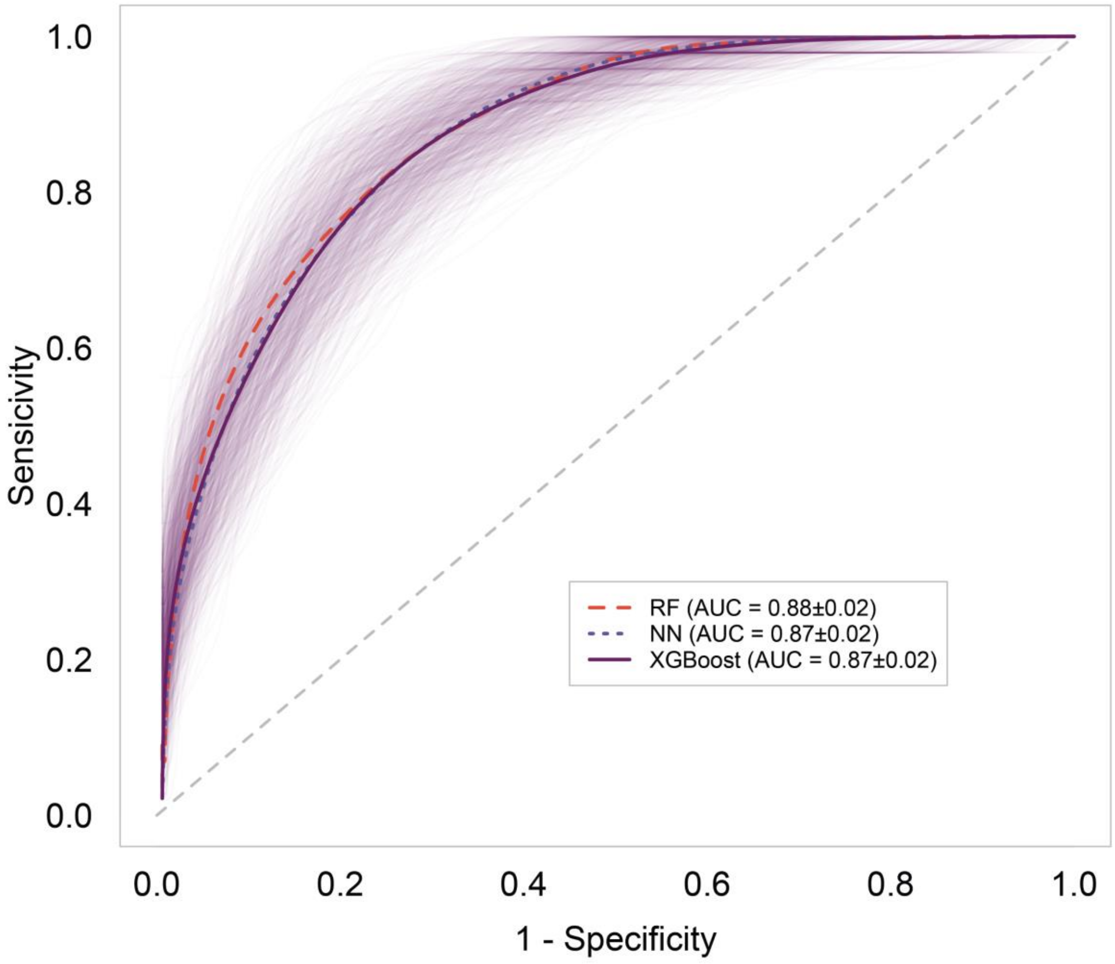

Figure S9. ROCs for XGBoost models in training dataset with 10-fold internal cross-validation repeated 100 times.

Abbreviations: ROCs, receiver operating characteristic curves; RF, random forest; NN, neural network; XGBoost, extreme gradient boosting.

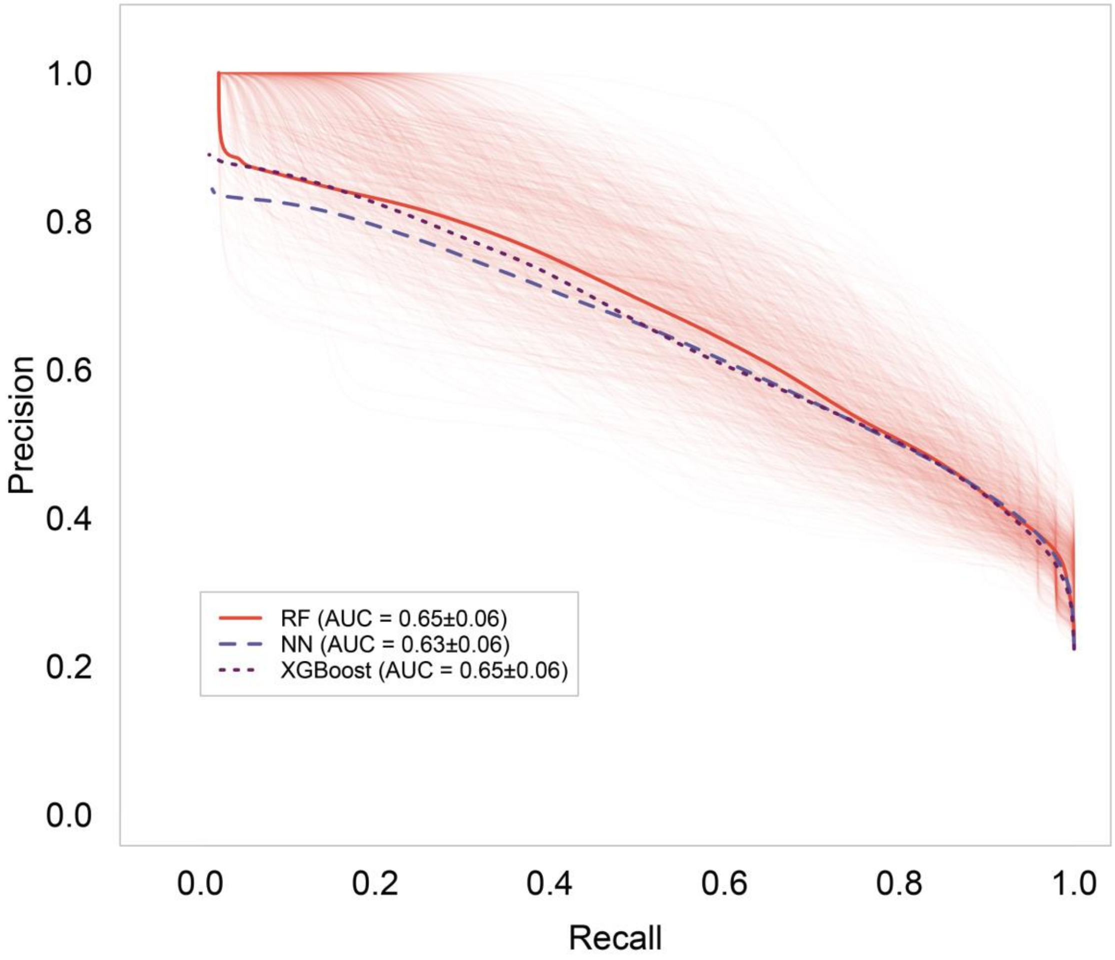

Figure S10. PRs for RF models in training dataset with 10-fold internal cross-validation repeated 100 times.

Abbreviations: PRs, precision-recall curves; RF, random forest; NN, neural network; XGBoost, extreme gradient boosting.

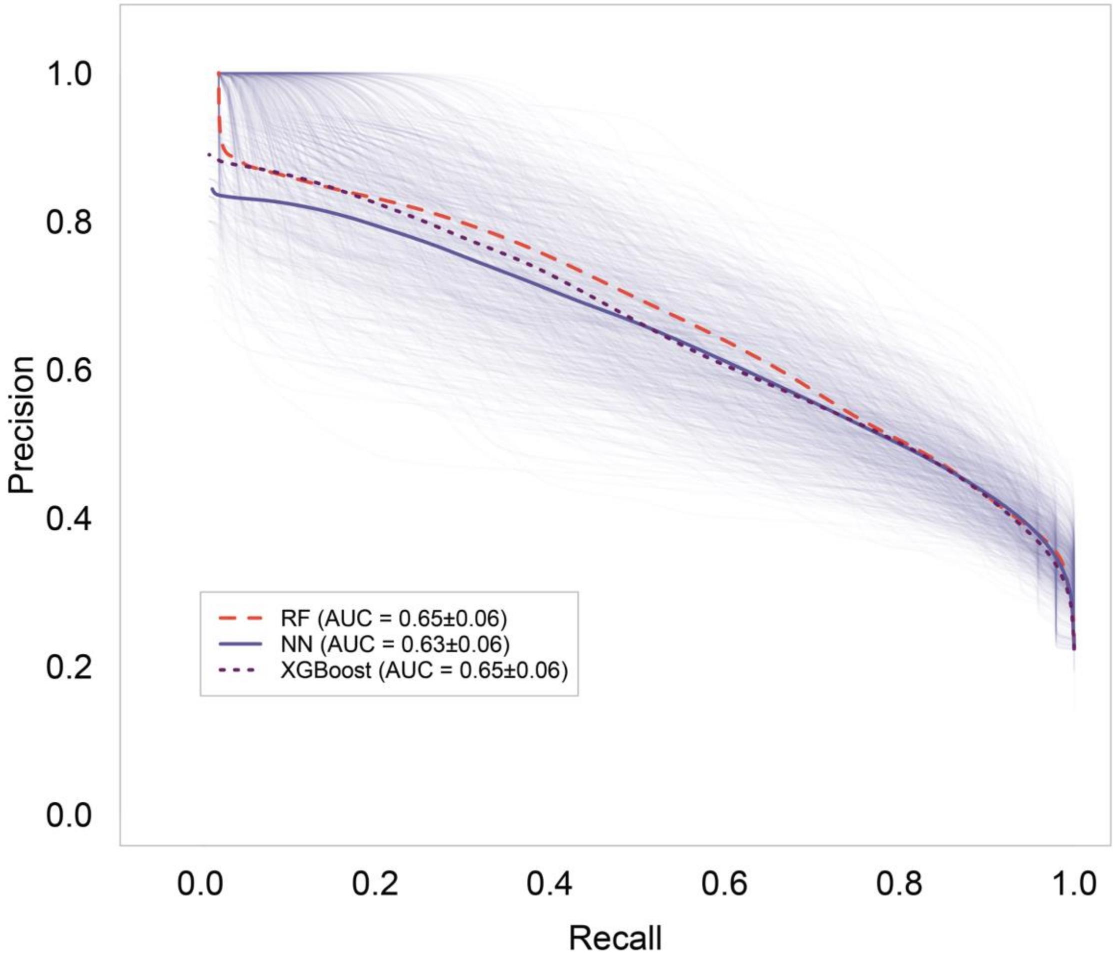

Figure S11. PRs for NN models in training dataset with 10-fold internal cross-validation repeated 100 times.

Abbreviations: PRs, precision-recall curves; RF, random forest; NN, neural network; XGBoost, extreme gradient boosting.

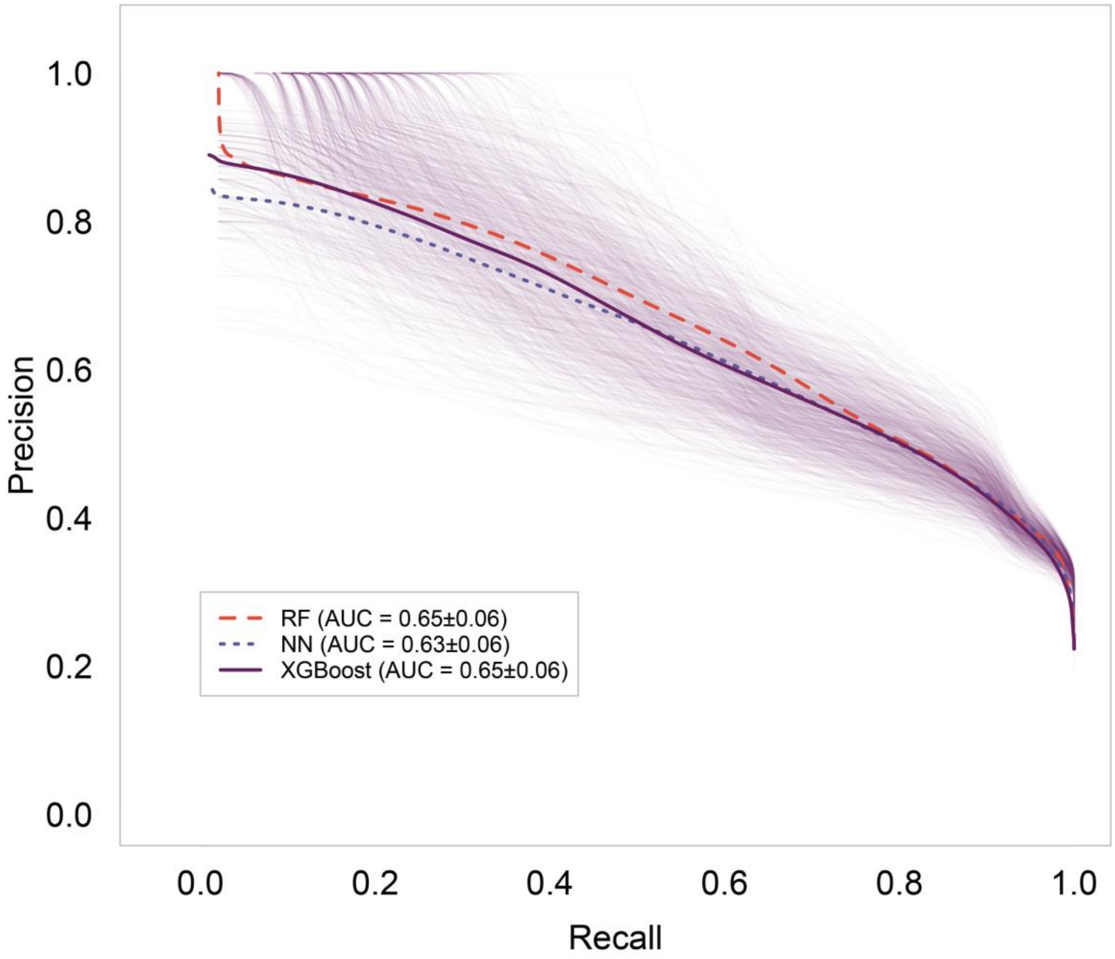

Figure S12. PRs for XGBoost models in training dataset with 10-fold internal cross-validation repeated 100 times.

Abbreviations: PRs, precision-recall curves; RF, random forest; NN, neural network; XGBoost, extreme gradient boosting.

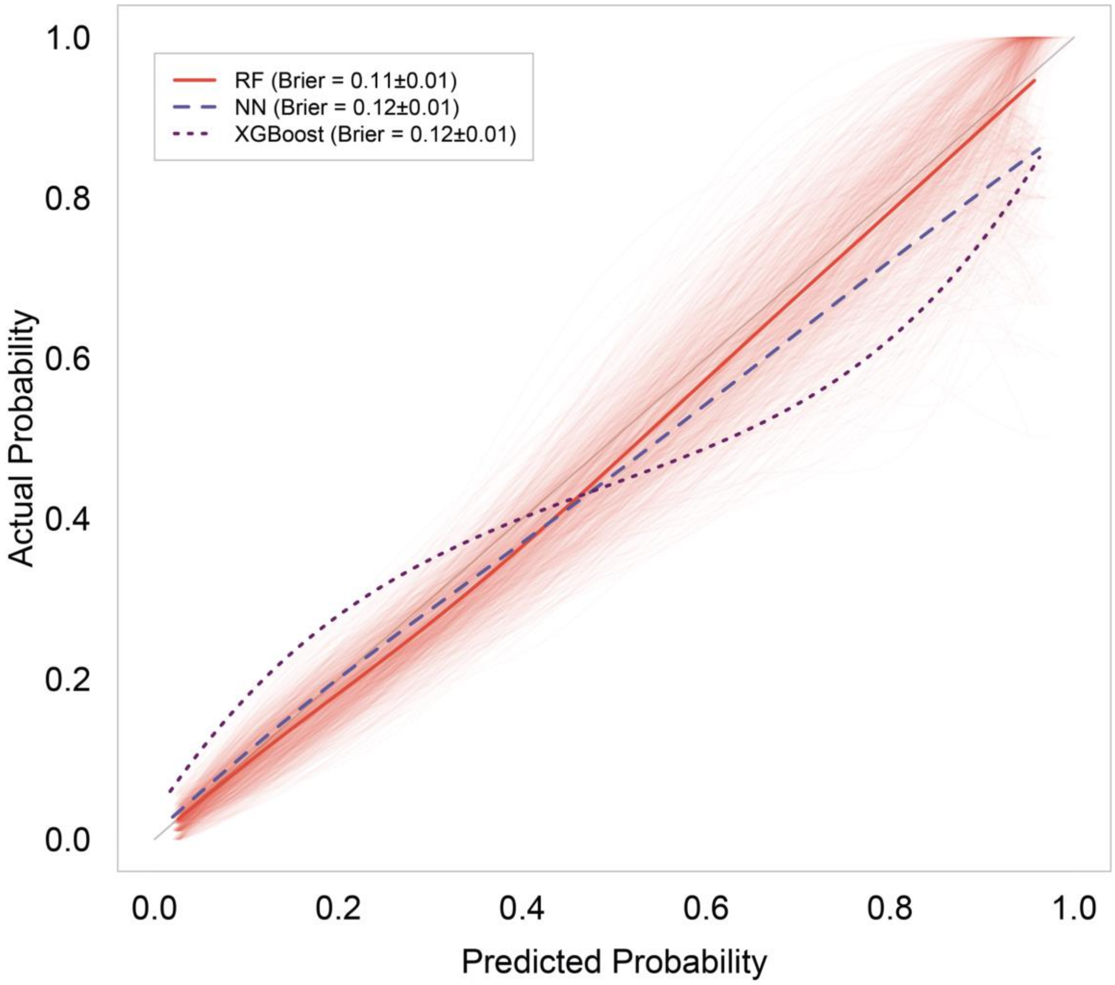

Figure S13. Calibrate plot**s** for RF models in training dataset with 10-fold internal cross-validation repeated 100 times.

Abbreviations: RF, random forest; NN, neural network; XGBoost, extreme gradient boosting.

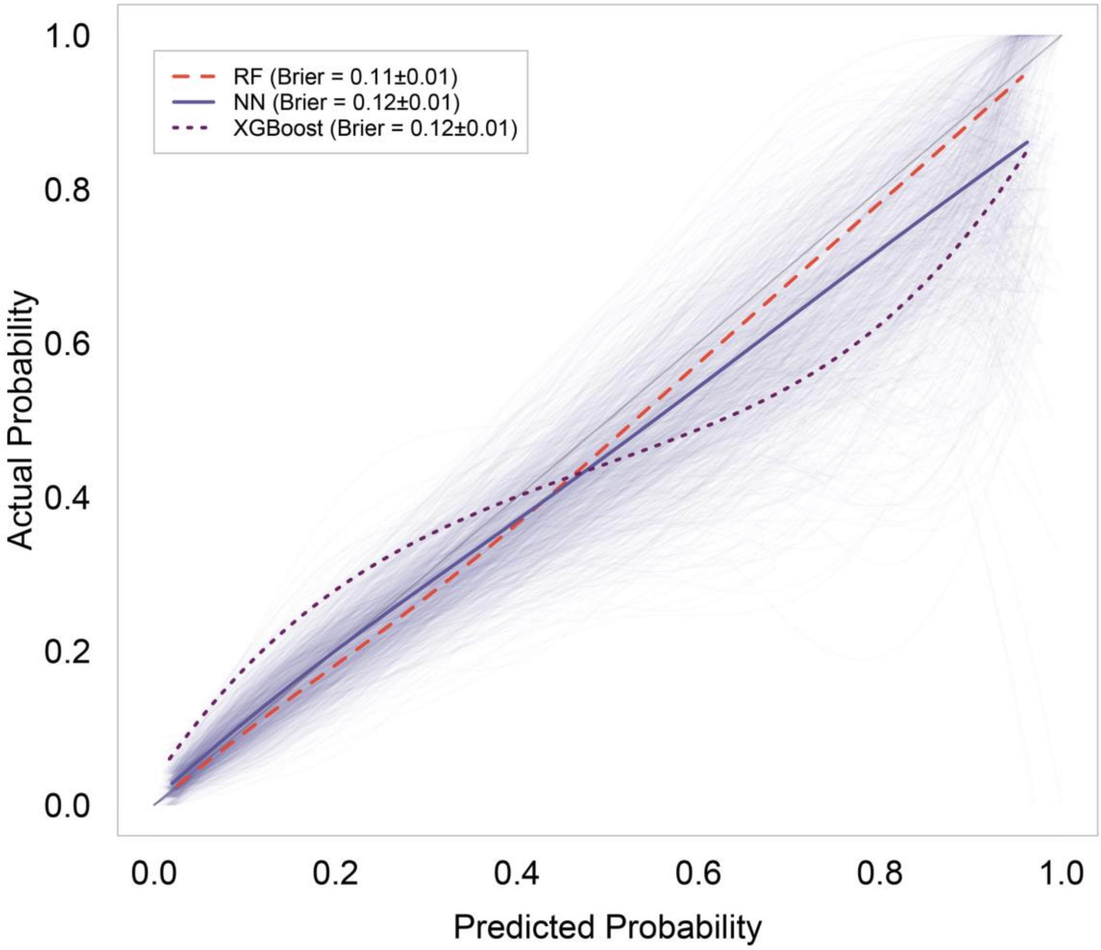

Figure S14. Calibrate plots for NN models in training dataset with 10-fold internal cross-validation repeated 100 times.

Abbreviations: RF, random forest; NN, neural network; XGBoost, extreme gradient boosting.

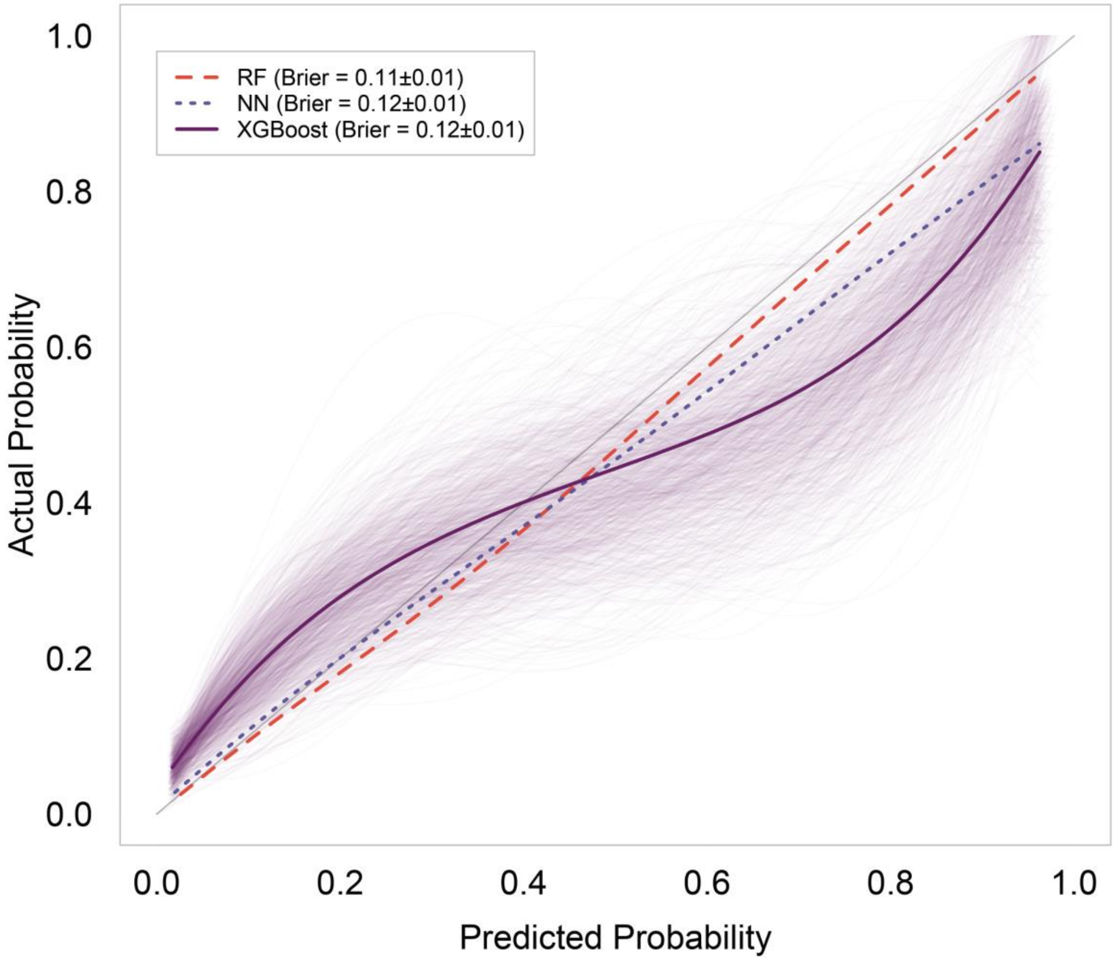

Figure S15. Calibrate plot**s** for XGBoost models in training dataset with 10-fold internal cross-validation repeated 100 times.

Abbreviations: RF, random forest; NN, neural network; XGBoost, extreme gradient boosting.

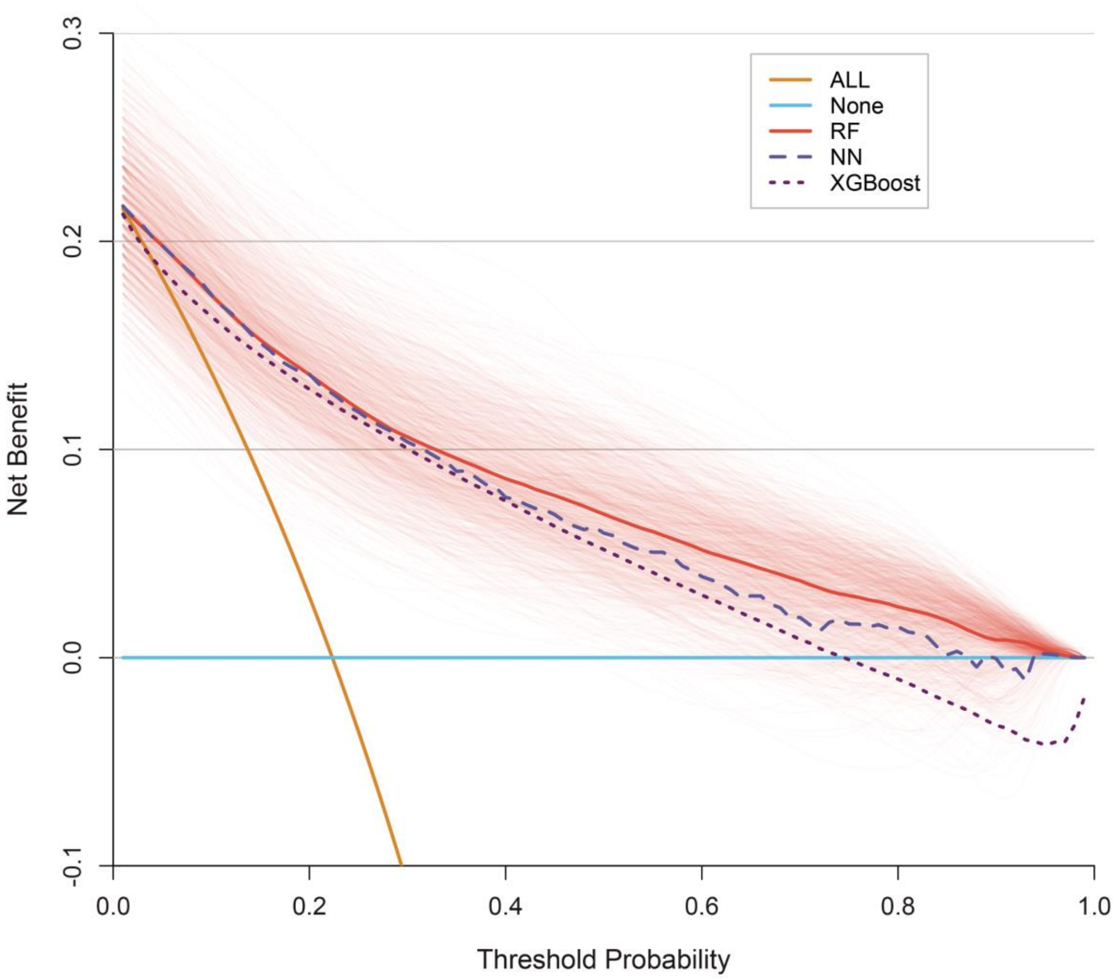

Figure S16. Decision curves for RF models in training dataset with 10-fold internal cross-validation repeated 100 times.

Abbreviations: RF, random forest; NN, neural network; XGBoost, extreme gradient boosting.

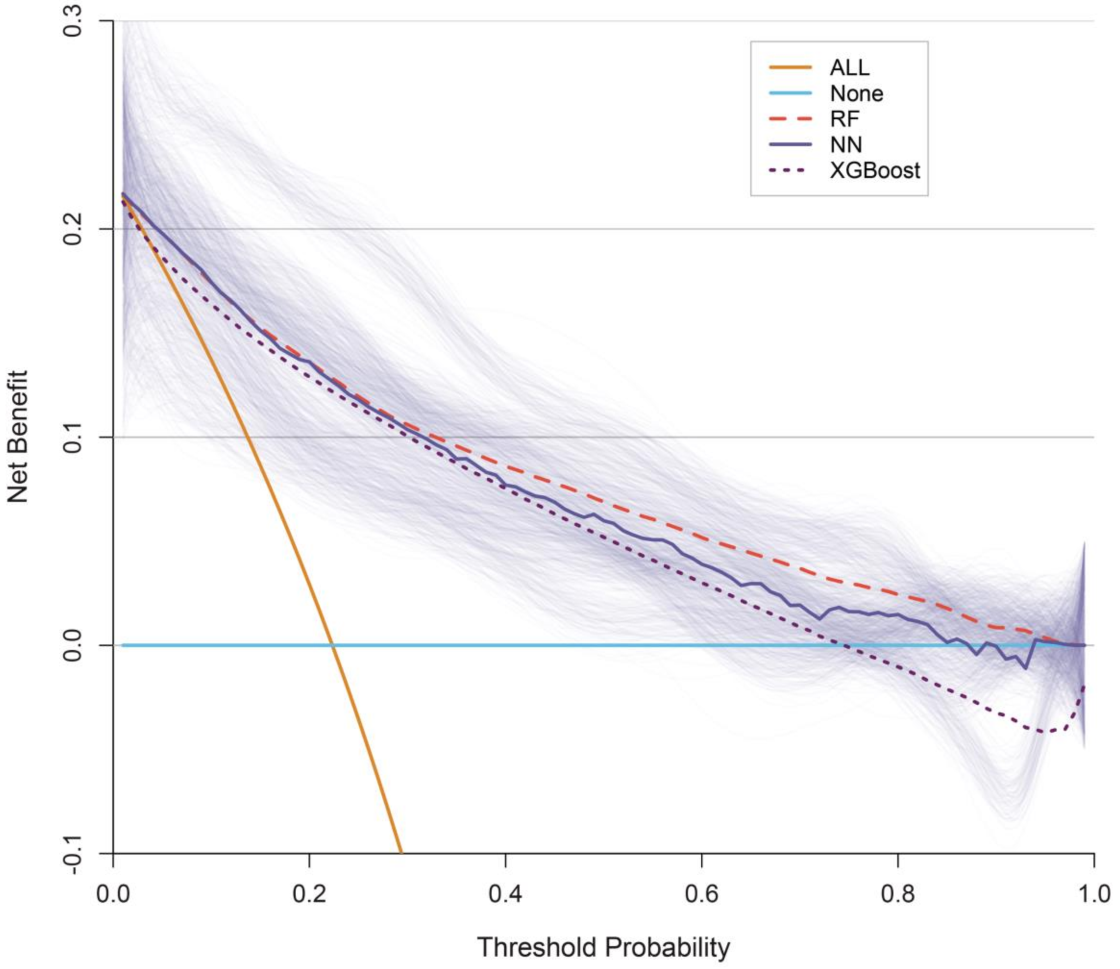

Figure S17. Decision curves for NN models in training dataset with 10-fold internal cross-validation repeated 100 times.

Abbreviations: RF, random forest; NN, neural network; XGBoost, extreme gradient boosting.

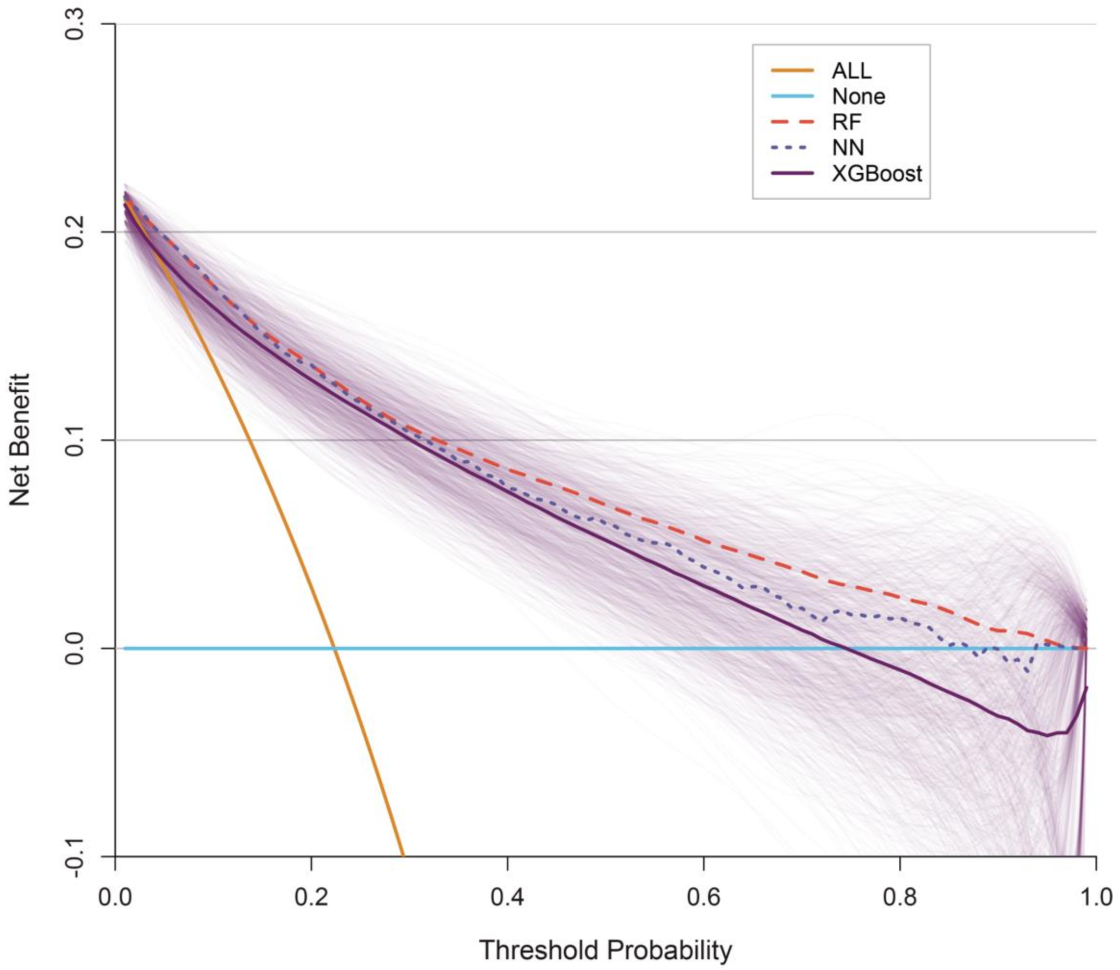

Figure S18. Decision curves for XGBoost models in training dataset with 10-fold internal cross-validation repeated 100 times.

Abbreviations: RF, random forest; NN, neural network; XGBoost, extreme gradient boosting.

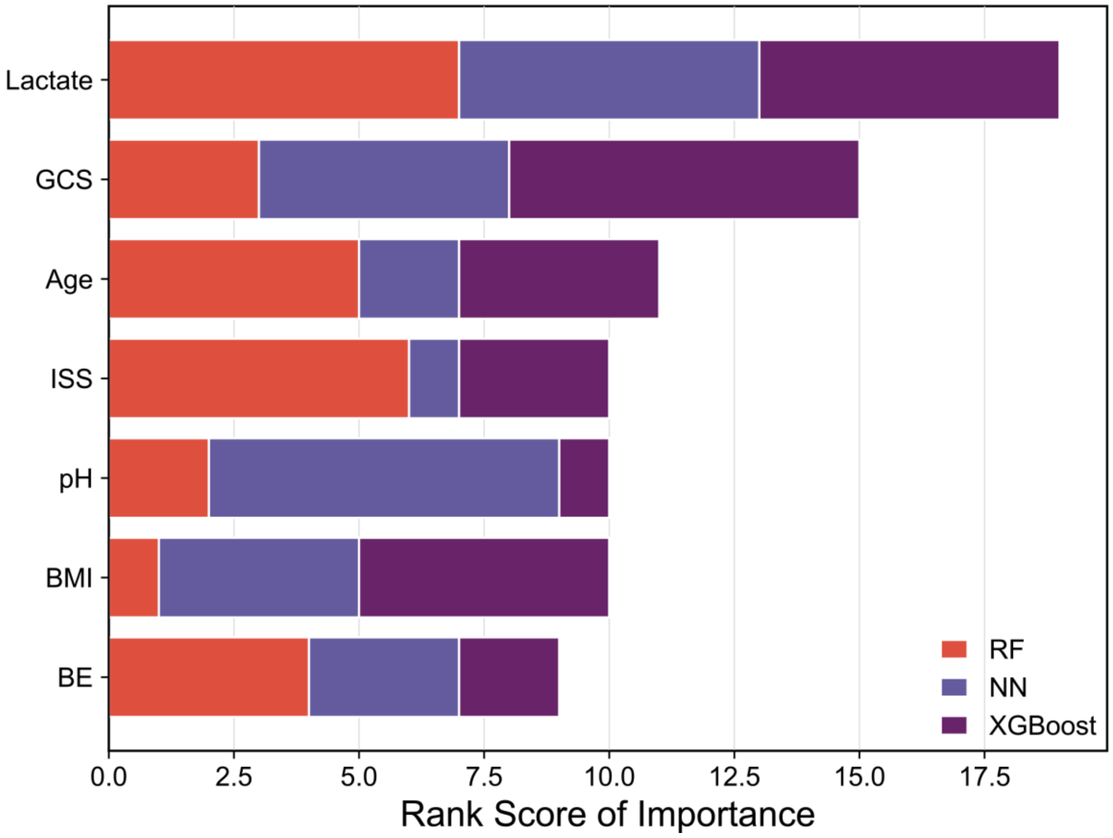

Figure S19. Rank score of importance for predictors.

Abbreviations: RF, random forest; NN, neural network; XGBoost, extreme gradient boosting; GCS, Glasgow coma scale; ISS, injury severity score; BMI, body mass index; BE, base excess.

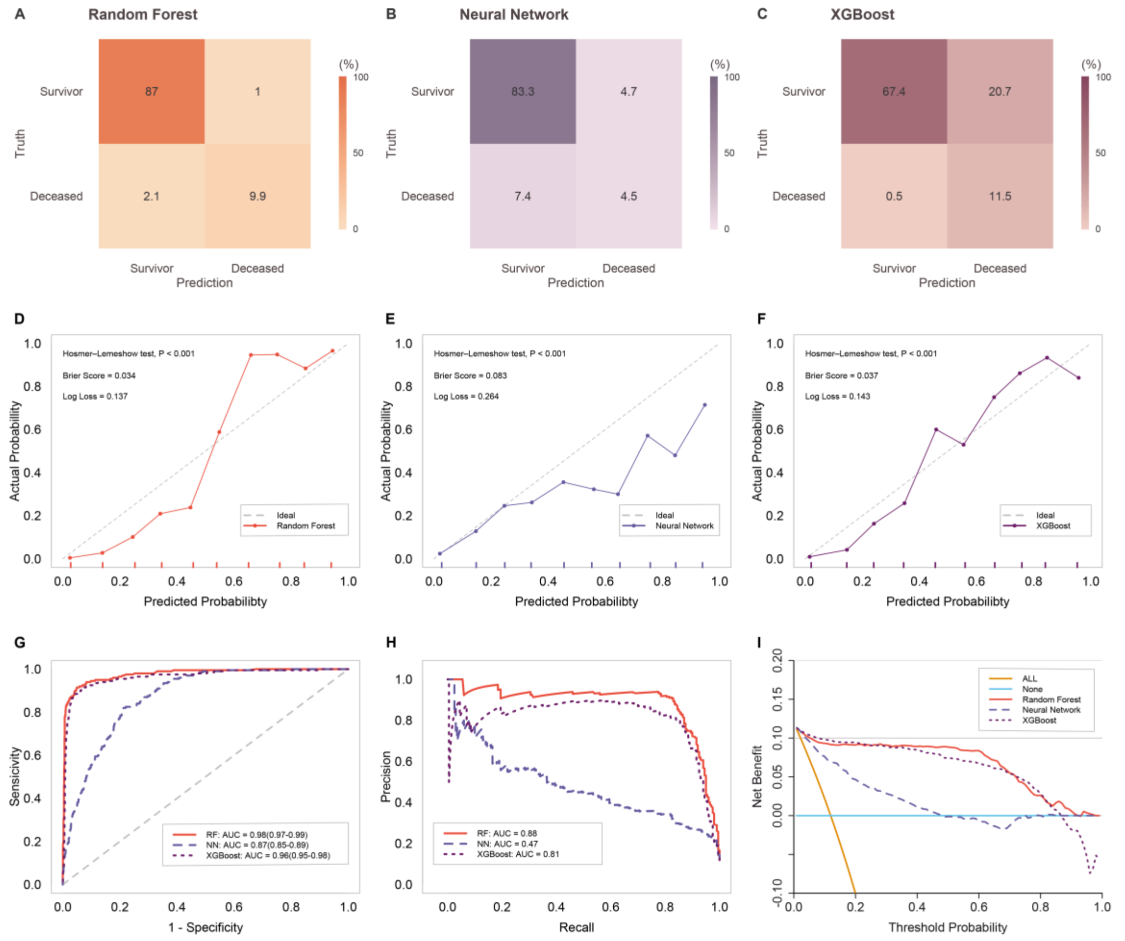

Figure S20. Confusion matrix plots, calibration plots, AUROCs, AUPRCs, and DCAs for models in the external validation cohorts.

Abbreviations: RF, random forest; NN, neural network; XGBoost, extreme gradient boosting; AUC, area under curve; AUROC, area under the receiver operating characteristic curve; AUPRC, area under the precision-recall curve; DCA, decision curve analysis.

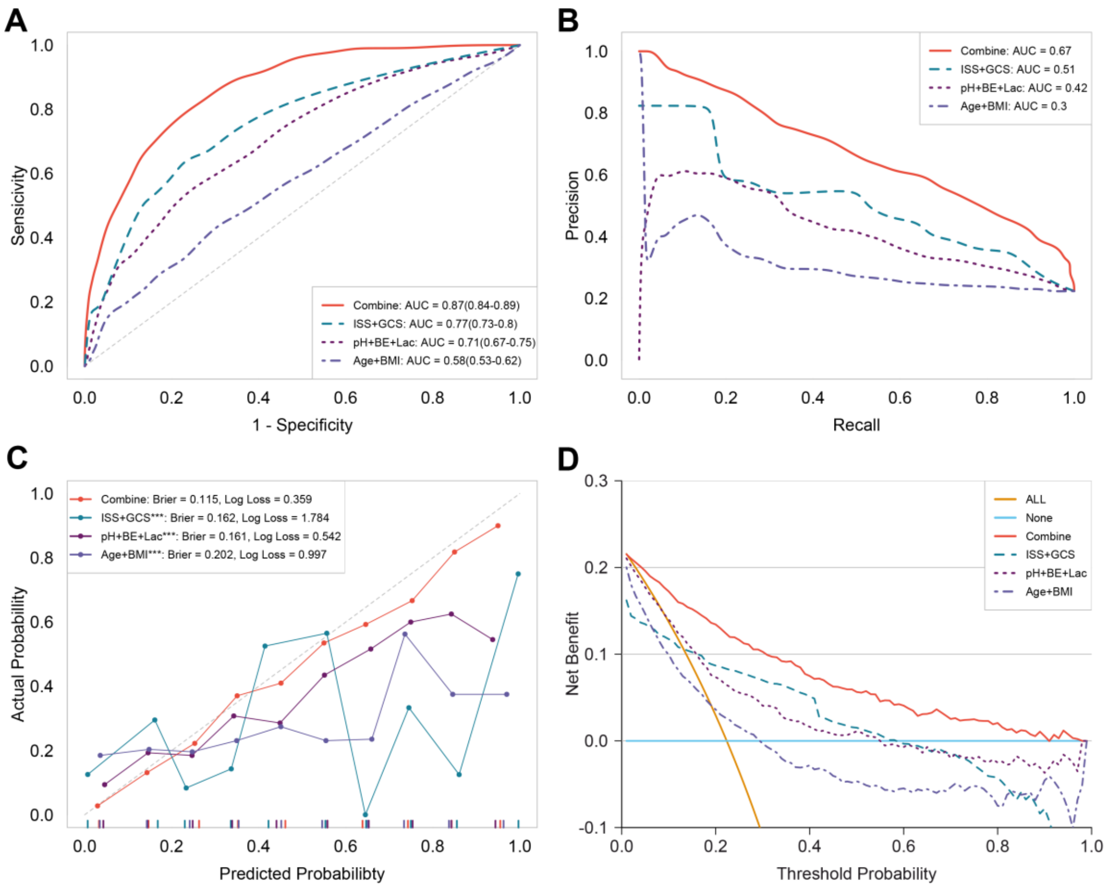

Figure S21. AUROCs, AUPRCs, calibration plots, and DCA for random forest models in the internal validation cohorts.

Abbreviations: AUROC, area under the receiver operating characteristic curve; AUPRC, area under the precision-recall curve; DCA, decision curve analysis; GCS, Glasgow coma scale; ISS, injury severity score; BMI, body mass index; BE, base excess. $^{***}P$ for Hosmer-Lemeshow test < 0.001.

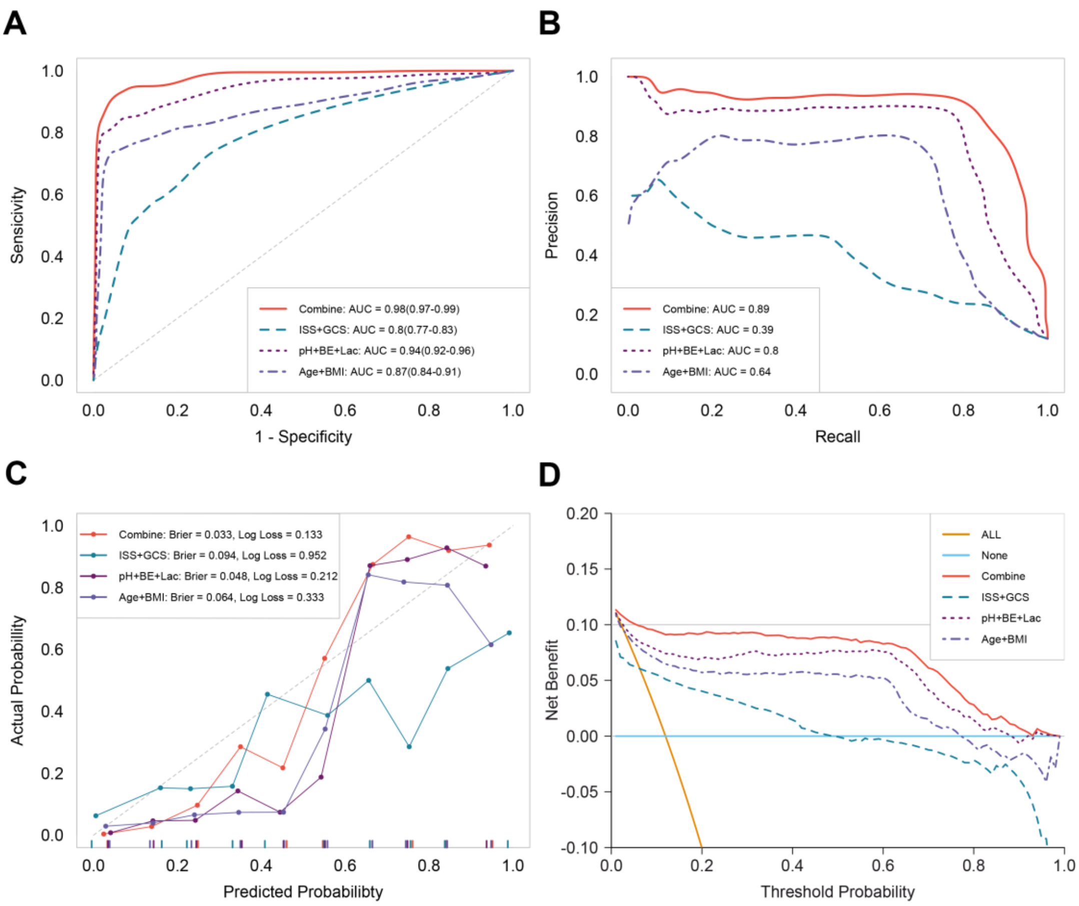

Figure S22. AUROCs, AUPRCs, calibration plots, and DCA for random forest models in the external validation cohorts.

Abbreviations: AUROC, area under the receiver operating characteristic curve; AUPRC, area under the precision-recall curve; DCA, decision curve analysis; GCS, Glasgow coma scale; ISS, injury severity score; BMI, body mass index; BE, base excess.
